# Characterization of *Cercospora nicotianae* Hypothetical Proteins in Cercosporin Resistance

**DOI:** 10.1371/journal.pone.0140676

**Published:** 2015-10-16

**Authors:** Aydin Beseli, Roslyn Noar, Margaret E. Daub

**Affiliations:** Department of Plant and Microbial Biology, North Carolina State University, Raleigh, North Carolina, United States of America; University of California, UNITED STATES

## Abstract

The photoactivated toxin, cercosporin, produced by *Cercospora* species, plays an important role in pathogenesis of this fungus to host plants. Cercosporin has almost universal toxicity to cells due to its production of reactive oxygen species including singlet oxygen. For that reason, *Cercospora* species, which are highly resistant to their own toxin, are good candidates to identify genes for resistance to cercosporin and to the reactive oxygen species it produces. In previous research, the zinc cluster transcription factor *CRG*1 (*c*ercosporin *r*esistance *g*ene 1) was found to be crucial for *Cercospora* species’ resistance against cercosporin, and subtractive hybridization analysis identified 185 genes differentially expressed between *Cercospora nicotianae* wild type (wt) and a *crg1* mutant. The focus of this work was to identify and characterize the hypothetical proteins that were identified in the *Cercospora nicotianae* subtractive library as potential resistance factors. Quantitative RT-PCR analysis of the 20 genes encoding hypothetical proteins showed that two, *24cF* and *71cR*, were induced under conditions of cercosporin toxicity, suggesting a role in resistance. Transformation and expression of *24cF* and *71cR* in the cercosporin-sensitive fungus, *Neurospora crassa*, showed that *71cR* provided increased resistance to cercosporin toxicity, whereas no significant increase was observed in *24cF* transformants. Gene disruption was used to generate *C*. *nicotianae 71cR* mutants; these mutants did not differ from wt *C*. *nicotianae* in cercosporin resistance or production. Quantitative RT-PCR analysis showed induction of other resistance genes in the *71cR* mutant that may compensate for the loss of 71cR. Analysis of 71cR conserved domains and secondary and tertiary structure identify the protein as having an NTF2-like superfamily DUF1348 domain with unknown function, to be intracellular and localized in the cytosol, and to have similarities to proteins in the steroid delta-isomerase family.

## Introduction

The purpose of this work is to identify genes encoding hypothetical proteins that may play a role in resistance of *Cercospora* fungi to the photoactivated toxin cercosporin produced by these fungi for infection of host plants. *Cercospora* species cause leaf spot and blight diseases on many major crops including corn, soybean, sugar beet, and coffee, leading to significant crop losses world-wide. One of the reasons for the success of these pathogens is their production of cercosporin, a photoactivated perylenequinone toxin [1]. Mutants deficient in cercosporin production are significantly less virulent on their host plants, thus engineering cercosporin-resistant crop plants via expression of cercosporin resistance genes may be an ecologically friendly method for controlling these damaging diseases.

Our work has focused on identifying cercosporin-autoresistance genes from *Cercospora* species, as cercosporin is almost universally toxic to cells due to its production of reactive oxygen species (ROS) [2]. In the light, cercosporin is converted to an energetically activated triplet state, which reacts with oxygen, generating ROS including singlet oxygen (^1^O_2_), a highly toxic species of ROS. Unlike free-radical forms of ROS that are components of cellular metabolism and for which defense mechanisms are understood, cellular resistance to ^1^O_2_ is not well characterized [2], and cercosporin is toxic to mice, bacteria, and many fungi in addition to host and non-host plants. During disease development, production of ^1^O_2_ and other ROS leads to peroxidation of cell membrane lipids in host plants and can also damage nucleic acids, proteins and lipids in the target cells [1]. *Cercospora* fungi are immune to cercosporin toxicity, thus they may be a source of genes for engineering crop resistance [3], and also serve as a model for understanding cellular resistance to ^1^O_2_.

Characterization of *C*. *nicotianae* mutants selected for sensitivity to cercosporin [4,5] led to the discovery of the zinc cluster transcription factor *CRG*1 (*c*ercosporin *r*esistance *g*ene 1) [6] required for autoresistance to cercosporin. To identify putative resistance genes, a subtractive cDNA library was generated between the *C*. *nicotianae* wild type and a *crg1* mutant [7]. From the library, 185 differentially regulated expressed sequence tags (ESTs) were found that are candidate resistance genes. The ESTs were classified into functional categories based on their homology to known sequences. These functional categories include ones known to be involved in cercosporin resistance including reductases [8], antioxidants and quenchers of ROS [9], and membrane transporters [10–13]. Several of the library genes have been characterized for their role in cercosporin resistance. For example, two genes encoding transporters in the library, *CnATR1* and *CnCFP*, were determined to have an important role in resistance [10,12] as disruption of either of these genes in *Cercospora* species caused the disruptant strains to be sensitive to cercosporin. In addition, tobacco transformed to express CFP showed significantly reduced lesion size after inoculation with *C*. *nicotianae*, confirming a role in resistance and suggesting that CFP might be useful for engineering plants for disease resistance [14]. In the case of ROS quenchers, vitamin B6 was shown to quench ^1^O_2_ and be involved in defense against cercosporin [9,15]. Studies to engineer tobacco to constitutively express *C*. *nicotianae* B6 biosynthetic genes (PDX1 and PDX2), however, resulted in no statistically significant increase in levels of the B6 vitamers, due to heavy regulation of these genes in plants [16]. Although antioxidants have been implicated in cercosporin resistance [2], a recent study of a gene encoding glutathione S-transferase from the library failed to demonstrate a link with cercosporin resistance [17].

In addition to genes that were categorized into defined functional categories, the library contains genes encoding hypothetical proteins whose functions are not yet characterized. Most studies of cellular resistance to ^1^O_2_ have been done with photosynthetic organisms, as ^1^O_2_ is a byproduct of photosynthesis [2]. In *Rhodobacter sphaeroides*, a model for studies of ^1^O_2_ resistance, resistance is associated with protein synthesis and turnover, amino acid metabolism, and glutathione-dependent and–independent detoxification pathways, among others [2]. A recent study comparing putative resistance genes from our *Cercospora* subtractive library with orthologs identified in transcriptional studies of *Rhodobacter* ^1^O_2_ resistance, however, showed little commonality between the two species [17]. Thus *Cercospora* fungi must have unique mechanisms not common to photosynthetic organisms. We thus chose to investigate the library genes encoding hypothetical proteins as possible novel sources of cercosporin and ^1^O_2_ resistance.

## Materials and Methods

### Culture conditions, fungal strains, and plasmids

Potato dextrose agar (PDA; Difco, Sparks, MD) was used for growth and maintenance of all *C*. *nicotianae* strains including the wild type (wt) strain ATCC18366. *Neurospora crassa* strains including the wt strain ORS-6a (Fungal Genetics Stock Center) were grown on Vogel’s medium [18].

DNA plasmid isolation, cloning, and ligation used standard molecular techniques [19]. iProof High-Fidelity DNA Polymerase from Bio-Rad Laboratories and OneTaq® DNA Polymerase from New England Biosciences were used along with the gene-specific primers (Tables 1 and 2) for standard PCR work. The plasmid pGEM-T Easy (Promega, Madison, WI) was used for cloning and sequencing. Plasmid pCB1636 [20] and pTxA-1 [12], were used for cloning, and fungal transformation. *Escherichia coli* strain DH5-α was used to maintain all plasmids.

**Table 1:** Primer sets used for RT-qPCR analysis for screening *C*. *nicotianae* hypothetical protein genes for induction under cercosporin toxicity conditions.

Gene	Forward Primer	Reverse Primer	Efficiency^1^
11sf	AGTGGCTAATCTGCTCTGG	CACTGCTATTCCTGTTGG	100.08
15cF	ACATGCCGCACTCTGTTCATTTGG	GAAGCCCACAACCTGCAACCATTT	104.48
1cF	CCGTTCCTGCAGAGCTCAAG	GCCATTGACCATGTTGAGCATGTC	109.33
200sF	TGCTGATCCGCTAAATGCTAAAAC	GCCGATGGACAAGGGTATAAGATC	100.21
207sF	ATGCTCGATCCTCCGAACCA	CGGCAGCTTTGAGCGTCTTT	101.78
214sR3	ATCAGAAGAGACAGCATAAAGC	AAGTGGTGGCTCAGCGTGG	89.57
24cF	TGCTTTCACCTTCAAGTTCGAC	TGCCCTTGCCAAAGCTAGG	106.51
40cR	TTGGTCCATCCCTGATCCTGTTGT	TCAGACTCAGCGAGCGAAGGATTT	99.25
55cR	GGAGACAGCCAAGCAAGAAGTC	GGGAAGAAGGCGATTGAGGA	100.16
56cR	ATCGTTCAAGACCGAGAGGCTCAA	ATGCCGAGAGATCAATGTCCCGAT	105.38
71cR	TCAAGCCACCCTACAATGCCTCAA	TTATTTGGTCGGTGCCTTGGACGA	100.09
77sR3	TGAGTGGTCGCTTGATTCG	ATCTGGACCCGAAATCGTGC	97.62
84sF	AGTGGGAGTGGGAGTCTTGG	AACACTGGAGAACGAATCAACG	91.29
Actin	TGACGATGCGCCACGAGCTGT	TTGATTGGAGCCTCGGTGAGC	101.20

^1^ Calculated efficiencies for primers for RT-qPCR analysis

**Table 2:** Primer sequences for gene cloning and for screening transformants^1^

Purpose	Primer Name	Sequence
ToxA plasmid cloning	71cR-F-EcoRI	TTTAAT**GAATTC**AGTGAACACGAACGACTAGGATG
ToxA plasmid cloning	71cR-R-HindIII	TTTAAT**AAGCTT**TCCACGATACGAACTAATGCTCACC
ToxA plasmid cloning	24cF-F-EcoRI	TTTAAT**GAATTC**TCGTAACATCGTTGGGTCAG
ToxA plasmid cloning	24cF-R-HindIII	TTTAAT**AAGCTT**TCGTAACATCGTTGGGTCAG
*N*. *crassa* transformant screening	71cR-F	GAGGAGGAGAGGTGGTTCAAGGA
*N*. *crassa* transformant screening	24cF-F	TTCCAATCTACGTTTTCGACCCTG
*N*. *crassa* transformant screening	Hyg-R	TGTCGGGCGTACACAAATCG
*N*. *crassa* transformant screening	ToxA-Rev1	ATAAAGGGCTAAGGTGTCCGTCC
*N*. *crassa* transformant screening	71cR-R	TTATTTGGTCGGTGCCTTGGACGA
*N*. *crassa* transformant screening	24cF-R	ATCAACTGGTAGGCGACTGTGAC
*71cR* disruption construct	71cF-5'-F ApaI	attaat**GGGCCC**TGCTCGTCATCTTGCTCATCG
*71cR* disruption construct	71cF-5'-R ApaI	attaat**GGGCCC**AGAGACATTTTGAGATGGAATTCG
*71cR* disruption construct	Hyg-split3S	CGTTGCAAGACCTGCCTGAA
*71cR* disruption construct	Hyg-split5A	GGATGCCTCCGCTCGAAGTA
*71cR* disruption construct	71cf-3'-R Sac1	attaat**GAGCTC**CAAGTCAAATCCAC
*71cR* disruption construct	71cF-3'-F Sac1	attaat**GAGCTC**GCCACCCTACAATGCCTCAAC
*71cR* disruption construct	71cF-5'-F	TGCTCGTCATCTTGCTCATCG
*71cR* disruption construct	71cf-3'-R	GAGCTCCAAGTCAAATCCAC
*C*. *nicotianae* disruption screening	71cR-Rev3	TCTTCCACTTCATCCCATCATTCTTA
*C*. *nicotianae* disruption screening	71cR-inv5’	CCTAGCATCTCAATCTCACCAACTAAC
*C*. *nicotianae* disruption screening	71cR-Forw10	CTAGATGAGACGACGCCTGATC
*C*. *nicotianae* disruption screening	Hyg-F2	TGAACCATCTTGTCAAACGACAC
*C*. *nicotianae* disruption screening	HygR	TGTCGGGCGTACACAAATCG
*C*. *nicotianae* disruption screening	71cR-DisR	GATGAAGTCGAGAGCACAACAAG

^1^ Bold and underlined sequences represent restriction enzyme targets

### Domains of hypothetical proteins in the library

The EST sequences recovered in the subtractive library between *C*. *nicotianae* wt and the *crg1* mutant [6] were used for finding orthologs of these EST sequences with full length sequence information using tBLASTx analysis against the NCBI (the US National Center for Biotechnology Information) protein database (11/21/2013 update). The full-length gene sequences of the orthologs were then used to find conserved domains using the Conserved Domain Database by NCBI.

### Gene expression under conditions of cercosporin toxicity

A *CnATR1* (*atr1*)-disrupted *C*. *nicotianae* mutant [12] and two *71cR*-disrupted *C*. *nicotianae* mutants (#16 and 18) were used to quantify gene expression under conditions of cercosporin toxicity as previously described [13]. RT-qPCR reactions were carried out as described [13]. The primers used to amplify each gene are shown in Table 1. Each sample was normalized against the *C*. *nicotianae*-specific actin reference gene, and fold-change relative to no-cercosporin (acetone control) was calculated according to the 2^−ΔΔ^C_T_ method [21–23].

### Sequencing of full length gene sequences of 24cR and *71cR*

Previously described methods [13] were used to obtain the full-length *C*. *nicotianae* genomic sequences of the two hypothetical protein genes, *71cR* and *24cF*, based on their partial EST sequences from the subtractive library [7]. Nucleotide and amino acid sequences of *71cR* and *24cF* are in the GenBank database with the accession numbers KJ126714 and KJ126715, respectively.

### Expression of hypothetical genes in *N*. *crassa*

The fungal transformation plasmid pTxA-1 [12], containing a hygromycin B (Hyg) resistance cassette and an ampicillin (Amp) cassette for selection in fungi and *E*. *coli*, respectively, was used to insert the two hypothetical protein genes, *71cR* and *24cF*, under the control of the fungal constitutive promoter *ToxA* from *Pyrenophora tritici-repentis*. Full-length copies of *71cR* and *24cF* were amplified, respectively, using primers 71cR-F-EcoRI and 71cR-R-HindIII (containing *EcoRI* and *HindIII* sites, respectively) for *71cR* amplification (512 bp) and 24cF-F-EcoRI and 24cF-R-HindIII (containing *EcoRI* and *HindIII* sites, respectively) for *24cF* amplification (1224 bp) (Table 2). PCR fragments were digested with *EcoRI* and *HindIII*, and ligated to the pTxA-1. The resulting plasmids, pTxA -71cR and pTxA -24cF, were sequenced to confirm the presence of an intact *71cR or 24cF* sequence.

The two plasmids were transformed into *N*. *crassa* as previously described [17]. Transformation and presence of *71cR* or *24cF* was confirmed in hyg-resistant colonies by PCR screening using gene specific primers (ToxA-Rev1 and 71cR-R, ToxA-Rev1 and 24cF-R, HYG-R and 71cR-F, HYG-R and 24cF-F) (Table 2). An NaOH DNA extraction method [24] was used to extract gDNA for PCR analysis.

### Screening of *N*. *crassa 71cR* and *24cF* transformants for cercosporin resistance

*N*. *crassa 71cR* and *24cF* transformants were tested for cercosporin resistance as previously described [17]. Percent growth on medium containing 10 μM cercosporin was calculated relative to the acetone control on the same plate by measuring radial growth of colonies at 21 hours after inoculation. Total RNA was extracted from 4 randomly chosen transformants for gene expression analysis, and RT-qPCR analysis was conducted as previously described [17]. The *N*. *crassa*-specific tubulin control was used as the reference gene.

### Disruption of *71cr* in *C*. *nicotianae*

The *71cR* gene was disrupted in wt *C*. *nicotianae* using a split-marker recombination technique previously described [25]. The 5’ (from 1517 bp upstream to 5 bp downstream of the start codon [1.5 kb]) and 3’ (from 443bp upstream to 43 bp downstream of the stop codon [0.5 kb]) *71cR* sequences were amplified from *C*. *nicotianae* gDNA by PCR with *71cR*-specific primers containing restriction enzyme linkers (Table 2). The two primer sets used to amplify the 5’ and 3’ ends of *71cR* were: 71cR-5’-F-*Apa*I, 71cR-5’-R-*Apa*I and 71cR-3’-F-*SacI*, 71cR-3’-R-*Sac*I. The PCR products were digested with the appropriate restriction enzymes (71cR-5’ digested with *Apa*I; 71cR-3’ digested with *Sac*I) for cloning into the receptor plasmid pCB1636 [20]. Plasmid pCB1636 was first digested with *Apa*I and ligated with *Apa*I digested *71cR*-5’. Recombinant plasmids isolated from this step were selected based on the insert’s correct orientation, and subsequently digested with *Sac*I and ligated with *SacI* digested *71cR*-3’ to obtain a *71cR* disruption construct. Using this construct as a template, two different overlapping PCR fragments were amplified using primers specific to the *71cR* sequence and the HygR cassette (split marker 1: 71cR-5’-F and HYG-split 5A [2.6 kb]; split marker 2: 71cR-3’-R and HYG-split 3S [1.3kb]) (Table 2). The identity of each of the split marker PCR fragments was confirmed by sequencing.

The methods used for isolation and transformation of the protoplasts of *C*. *nicotianae* were as previously described [13]. After transformation, colonies were transferred at least 5 times to PDA amended with 125 μg/ml hyg to ensure stability of transformation. *71cR* disruption was confirmed by PCR analysis using primer sequences shown in Table 2.

### Screening of *71cR* disruption mutants for cercosporin sensitivity and production

*71cR* disruptants confirmed with the PCR analysis described above were screened for cercosporin sensitivity as previously described [13]. Data shown are the result of two independent experiments with 4 replications each. Cercosporin production by each disruptant was measured at 480 nm as previously described [13,26].

### Phylogenetic analysis and species distribution of 71cR orthologs

Phylogenetic analysis of protein sequences was done with representatives from the DUF1348 family and each of the characterized families within the NTF2-like superfamily. The conserved NTF2-like domain from each sequence was identified using the Conserved Domain Database, and the domain sequences were aligned using the MUSCLE [27] algorithm in Mesquite v3.0.1 [28]. The protein substitution model WAG+G+F [29] was selected using ModelGenerator v0.85 [30]. The alignment was analyzed using maximum likelihood with raxmlGUI v. 1.3.1 [31] with no outgroup, using bootstrap analysis with the autoMRE function.

Blastp and tblastn searches were performed on the NCBI database (11/21/2013 update) to identify 130 of the closest orthologs of 71cR. The *Cercospora canescens* whole genome sequence (Accession number: ANSM00000000) was downloaded from NCBI, and tblastn in BLAST+ [32] was used to identify its 71cR ortholog (S1 Table). To further characterize the species distribution of 71cR orthologs, tblastn searches were done of the 71cR protein sequence against filtered model transcripts of each fungal genome available on the JGI Mycocosm portal [33,34].

### Secondary and tertiary structure prediction of 71cR

The secondary and tertiary structure of the 71cR amino acid sequence was analyzed using Raptor software (8/12/2015 update) [35]. Raptor software was also used for the ligand binding predictions. The Dali server (2/20/2014 update) [36] was used to find orthologs based on the tertiary structure similarities. The crystal structure of 71cR is not available, thus the crystal structure of the protein PFL_3262 from *Pseudomonas fluorescens* (PDB ID: 2IMJ) (the closest ortholog with a crystal structure), was used for the Dali analysis. Predict Protein server [37] was used to identify transmembrane helices or disulphide bridges and localization predictions for 71cR.

Conserved regions of 71cR were found by aligning 71cR with the closest 130 orthologs in S1 Table, using MUSCLE in Mesquite as described previously, and Chimera 1.10.1 [38] was used to display the degree of conservation for each residue within the conserved domain. These conserved residues were then compared with the ligand binding predictions from Raptor software.

### Assays for cercosporin modification or degradation

To test the activity of the 71cR protein to degrade or modify cercosporin, two methods were used. To assay for possible degradation of cercosporin, 10 mg of freshly growing mycelia of wt *N*. *crassa* and two *71cR N*. *crassa* transformants (# 379, 380) were collected and grown in 20 ml of PDB medium for 5 days in the dark at room temperature. Cercosporin was added to final concentration of 10 μM and incubated in the dark for 5 hours. To extract cercosporin from the mycelium, it was filtered and ground in liquid nitrogen, and cercosporin was extracted in acetone. The amount of cercosporin in the growth medium and in the mycelial extracts was analyzed by measuring the absorbance at 460 nm using a spectrophotometer. To assay for possible modification of cercosporin, mycelium of wt *N*. *crassa* and *71cR* transformants #379 and 380 was grown in PDB, and cultures were incubated for 5 hours with cercosporin at a final concentration of 10 μM. We imaged the mycelia using Zeiss LSM 710 confocal microscopy with a 488 nm argon laser reflecting off a 488 nm main beam splitter. A series of images with the same field of view at different emission wavelengths (λ-stack) was captured between 504 and 728 nm in 10 nm intervals. Images were analyzed offline using Zeiss ZEN software to generate the fluorescence emission spectra for the hyphae.

## Results

### Identification of genes encoding hypothetical proteins and domain analysis

To identify genes encoding resistance to the toxin cercosporin and to ^1^O_2_, we have been characterizing genes in a subtractive library [7] between the *C*. *nicotianae* cercosporin-resistant wt and the sensitive *crg1* mutant deficient for the CRG1 transcription factor [6]. In previous studies we characterized genes encoding membrane transporters [12,13] as well as enzymes that generate reducing power [17], as both toxin transport and toxin reduction have been identified as important mechanisms of cercosporin resistance [2]. Our focus in this study is on genes encoding hypothetical proteins, as resistance to ^1^O_2_ is poorly understood. We screened 13 ESTs from the library that are homologous to genes encoding hypothetical proteins. A genome sequence for *C*. *nicotianae* is not available. Therefore, tBLASTx searches of the EST sequences were performed against NCBI’s protein database to identify the closest orthologs. Functional domains of these orthologs were then identified using NCBI’s conserved domain database [39]. With the exception of 56cR, 40cR, and 200sF EST sequences, all EST sequences were aligned with their orthologs with high similarity (p-value of e^-10^ or lower).

The functional domains of the orthologs of the hypothetical EST sequences are listed in Table 3. No functional domains were found for the orthologs of 40cR or 77sR3 EST sequences with the p-value threshold of e^-5^. Orthologs of the remaining 11 hypothetical proteins had domains with characterized functions, some of which are consistent with previously identified mechanisms of resistance to cercosporin or ROS damage including reducing activity, amino acid turn-over, and transporters [2, 8, 12, 13, 14]. ESTs with these functions include 56cR, which has a hydrolase domain, 1cF and 200sF with domains involved, respectively, in tryptophan and tyrosine biosynthesis, and 15cF that has a permease transporter domain. Other identified domains include a phospholipid methyltransferase domain (24cR and 84cR), a peptidoglycan binding domain (11sf), domains for proteins triggering apoptosis (55cR), and ones involved in signal transduction (207sF).

**Table 3:** Conserved domains of the orthologs of hypothetical proteins in the subtractive library.

EST^1^	Domain Name^2^	Accession^3^	Description^4^	Interval^5^	E-value^6^
11sF	LysM	cd00118	Lysine Motif	130–258	6.18E-11
	LytE	COG1388	FOG: LysM repeat (found in outer membranes)	382–657	1.11E-08
	FAM53 super family	cl21101	Family of FAM53	170–514	4.44E-05
1cF	RHOD	cd00158	Rhodanese Homology Domain	1057–1404	3.08E-09
	Trp-synth-beta_II super family	cl00342	Tryptophan synthase beta superfamily (fold type II)	85–969	9.42E-40
56cR	Abhydrolase_6	pfam12697	Alpha/beta hydrolase family	241–1038	4.14E-16
	TT_ORF1 super family	cl20238	*Torque teno* viral orf 1	797–916	2.67E-05
15cF	RhaT	COG0697	Permeases of the drug/metabolite transporter (DMT) superfamily	461–1243	2.35E-14
24cF	PEMT super family	cl21511	Phospholipid methyltransferase	85–675	6.50E-38
55cR	EI24	pfam07264	Etoposide-induced protein 2.4	247–807	3.53E-10
71cR	NTF2_like super family	cl09109	Nuclear transport factor 2 (NTF2-like) superfamily	90–455	2.19E-54
84cR	PEMT super family	cl21511	Phospholipid methyltransferase	163–1020	1.05E-44
200sF	Tyrosinase	pfam00264	Common central domain of tyrosinase	295–984	2.98E-71
207sF	LDB19 super family	cl15231	Arrestin N terminal like family	578–1117	9.96E-39
214sR3	R3H-assoc	pfam13902	R3H-associated N-terminal domain	247–582	1.63E-34
40cR	no domains				
77sR3	no domains				

^1^ The library names of the EST sequences in the subtractive library [7]; F = forward library (down-regulated in *crg1* mutant); R = reverse library (upregulated in *crg1* mutant)

^2^ The domain name given in The Conserved Domain Database in the NCBI website [39]

^3^ Accession numbers of the conserved domains

^4^ A brief description of the domains

^5^ The positions within the *C*. *nicotianae* genes that the domains are aligning.

^6^ E-value indicates statistical significance for the similarity between the hypothetical protein sequence and the sequence of the characterized domain.

### Expression of hypothetical genes under conditions of cercosporin toxicity

**Fig 1:**
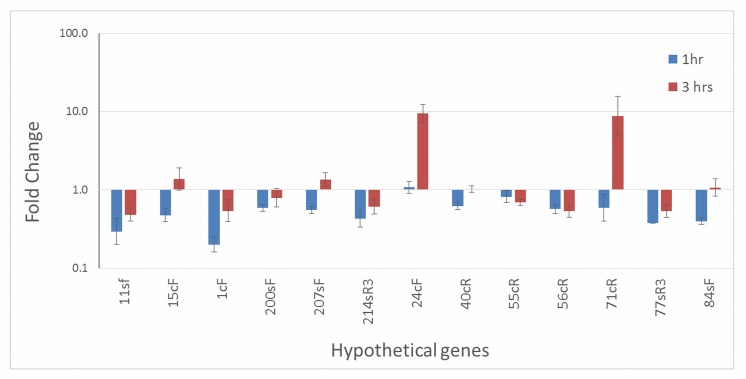
Quantitative RT-PCR analysis of gene expression of hypothetical protein genes in the *C*. *nicotianae atr1* mutant treated with cercosporin in the light. Each sample was normalized against the actin reference gene, and fold-change relative to no-cercosporin was calculated according to the 2^(-ΔΔC(T))^ method [21]. Data represent the mean of two independent experiments with three replications each. Error bars represent 95% confidence intervals.

In order to define a possible role in cercosporin resistance, the 13 genes encoding hypothetical proteins were assayed for changes in expression under conditions of cercosporin toxicity. The cercosporin-sensitive *C*. *nicotianae atr1* mutant, deficient in the ATR1 ABC transporter involved in cercosporin resistance [12], was treated with cercosporin under high-light conditions to induce toxicity, and gene expression was assayed by RT-qPCR. This mutant was used because it is highly sensitive to cercosporin and has previously been shown to be useful for identifying putative cercosporin-resistance genes that are upregulated under cercosporin toxicity conditions [13]. Genes *24cF* and *71cR* were upregulated 9.4- and 8.7-fold, respectively, three hours after cercosporin toxicity was induced (Fig 1). None of the other 11 genes were upregulated under these conditions. Based on these gene expression results, *24cF* and *71cR* were chosen for further characterization.

### Ability of 24cF and 71cR to impart resistance in *N*. *crassa*

**Fig 2:**
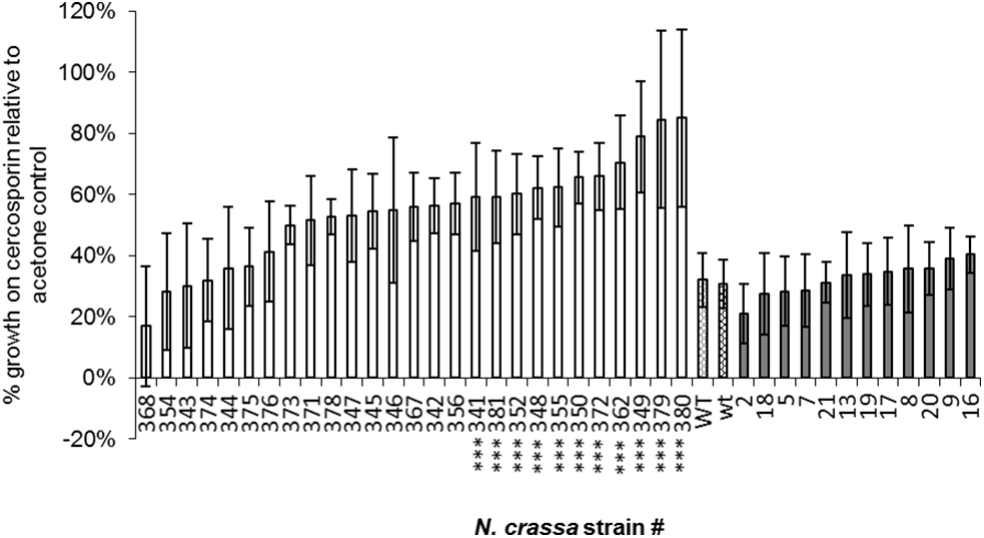
Cercosporin resistance of *Neurospora crassa 71cR-*transformed strains. White bars: *71cR* transformed strains; patterned bars: wild type *N*. *crassa* (WT = 71cR control; wt = 24cF control); grey bars: *24cF*-transformed strains. Data are the result of two independent experiments with 5 replications each. Strains marked with *** have significantly greater resistance than wild type (P < 0.05). Error bars represent 95% confidence intervals.

The cercosporin-sensitive fungus *N*. *crassa* was transformed with *24cF* and *71cR* to test the ability of these genes to impart cercosporin resistance. *N*. *crassa* was chosen for this assay as it is highly sensitive to cercosporin, easily transformable, and has been shown in previous studies as an excellent system for screening genes that impart cercosporin resistance [13, 17]. Hyg-resistant colonies after transformation were screened for the presence of the genes by PCR. Out of a total of 84 and 93 hyg-resistant colonies, respectively, of *71cR* and *24cF* transformants, 60 and 12, respectively, were confirmed to contain the intact transgene by PCR screening (data not shown). All 12 of the *24cF* and 27 of the *71cR* PCR-positive strains were assayed for resistance to cercosporin by measuring radial growth on cercosporin-containing medium relative to growth on control medium. Cercosporin at 10 μM inhibits radial growth of *N*. *crassa*, resulting in approximately 30% of the radial growth on medium lacking cercosporin (Fig 2 and S1 Fig). Of the randomly chosen 27 PCR-positive *71cR* transformants tested, eleven were found to be significantly more resistant to cercosporin than wt (P < 0.05) (Fig 2). However, none of the 12 PCR-positive *24cF* transformants tested had significantly greater resistance to cercosporin than wt (P < 0.05) (Fig 2).

**Fig 3:**
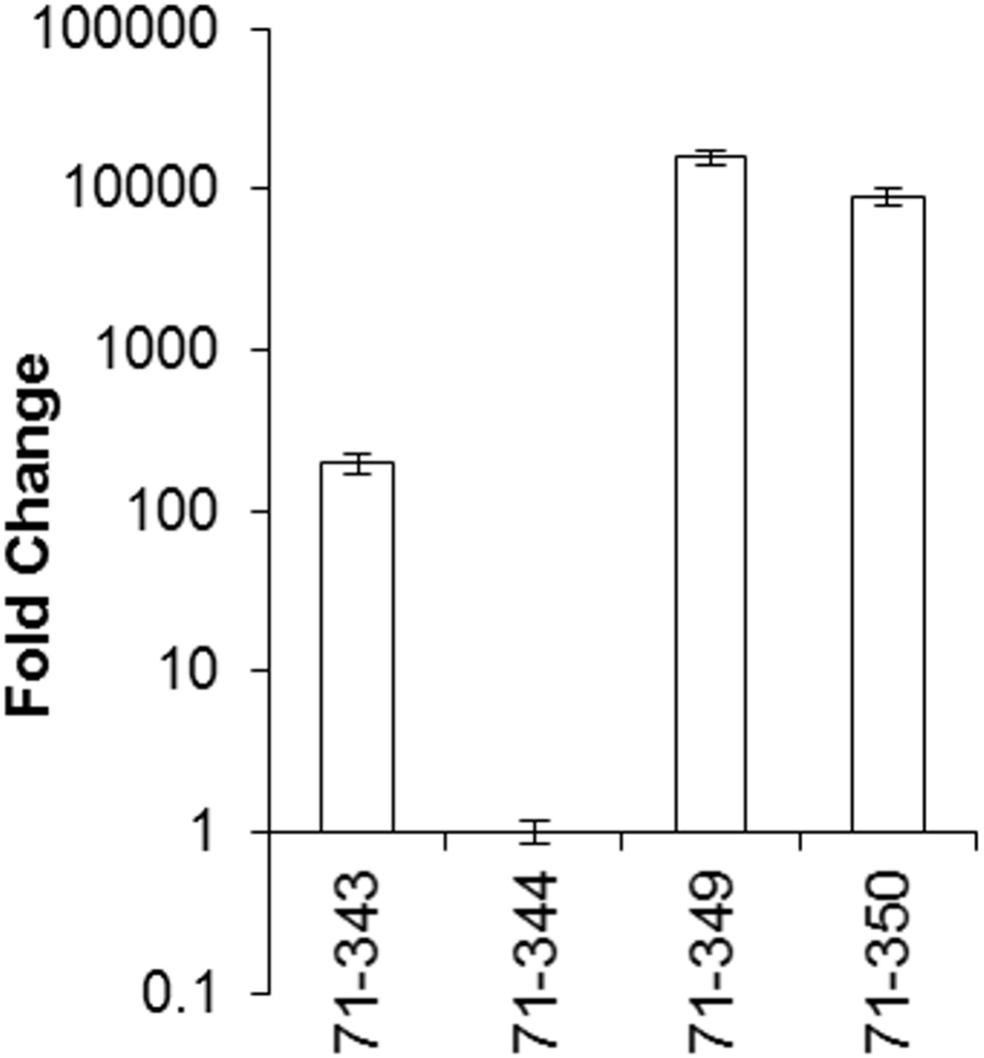
Quantitative RT-PCR analysis of gene expression of the *71cR* gene in selected *Neurospora crassa* transformants. Each sample was normalized against the tubulin reference gene, and fold-change relative to expression of the lowest expressing *Neurospora crassa* transformant was calculated according to the 2^(-ΔΔC(T))^ method. Error bars represent 95% confidence intervals.

Expression of the transgenes was assayed in selected transformants screened for resistance. RT-qPCR analysis of *71cR* expression in the two resistant *71cR* transformants assayed (#349 and 350) showed high levels of expression (15,973- and 9,006- fold increase respectively), compared to the lowest expresser, a non-resistant *71cR* transformant (#344) (Fig 3). Expression of the other non-resistant *71cR* transformant was much lower than the two resistant *71cR* transformants (199-fold change increase relative to #344). Thus *71cR* expression correlated with cercosporin resistance. For *24cF*, all four transformants tested had similar C_T_ values as the high-expressing *71cR* transformants, indicating expression of *24cF*, and there was little difference in expression between the four *24cF* transformants (data not shown). From these results we concluded that the 71cR protein can provide resistance to cercosporin toxicity, but that 24cF cannot.

### Disruption of *71cR* in *C*. *nicotianae* and phenotype of disruption mutants

**Fig 4:**
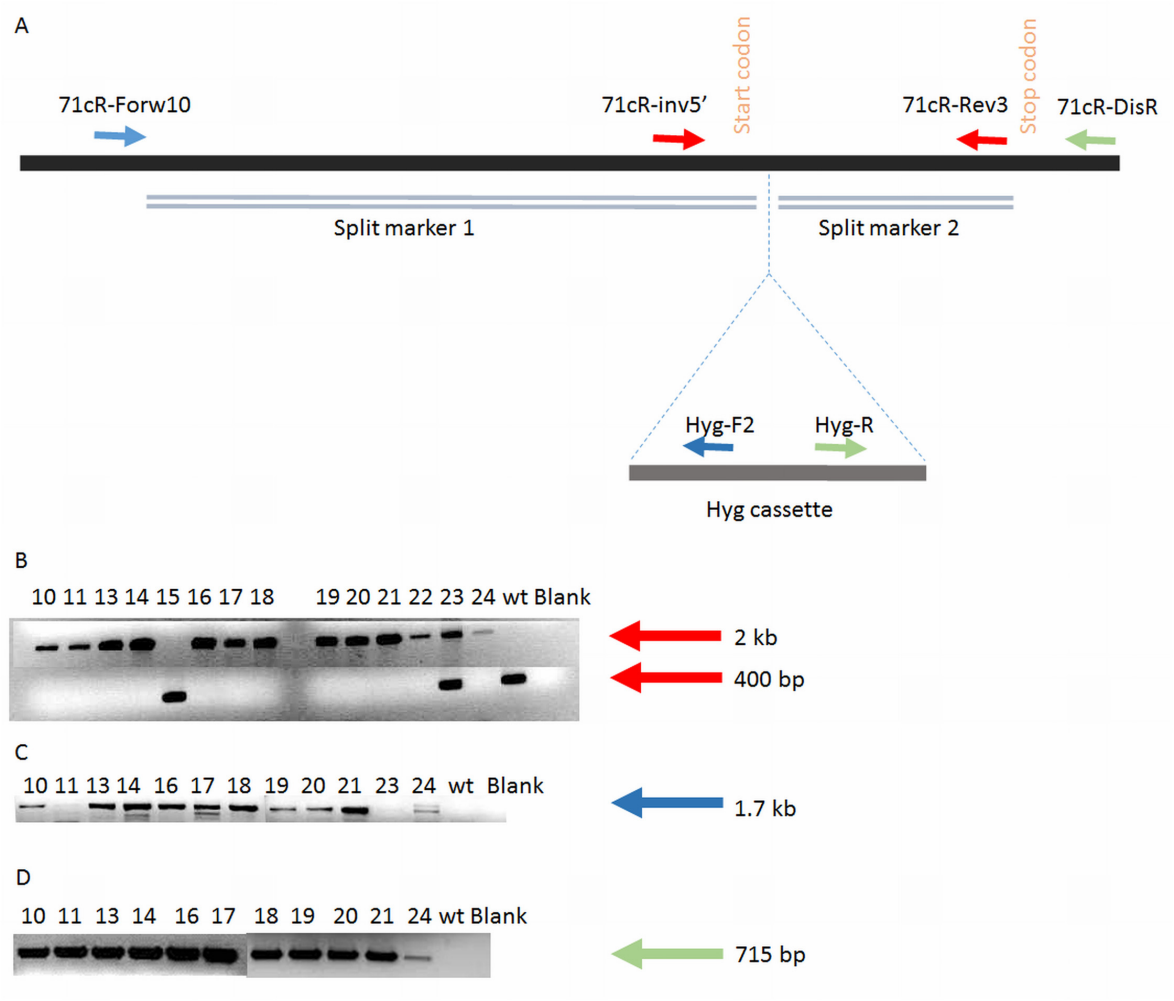
Disruption of *71cR*. **A**. The gDNA of wt *C*. *nicotianae* is shown as a black bar with the start and stop codons of *71cR* indicated. In disrupted transformants the hyg cassette is predicted to integrate in the location specified by dotted lines. The homologous regions of each split marker are represented as double lines. Location of primers used in PCR screening is also shown. **B**. Gel image of screening putative *71cR* disruptants with primers that span the hyg cassette integration site (71cR-Rev3 and 71cR-inv5’; red arrows in panel A). Integration results in a 2 kb band; wt band is 400bp (indication of non-disruptant genotype). Wild type *C*. *nicotianae* and transformant #15 showed only the wild type band; transformant #23 has both bands indicating an ectopic integration of the split markers. **C.** Bands represent the 1.7 kb amplification of the 5’ integration site by using a *71cR* locus-specific forward primer outside the split marker 1 sequence (71cR-Forw10) and a reverse primer specific to the hyg cassette sequence (Hyg-F2) (blue arrows in panel A). **D.** Bands represent the 715 bp amplification of the 3’ integration site (Hyg-R and 71cR-DisR; green arrows in panel A).

A split marker strategy was used to generate *C*. *nicotianae* disruption mutants by homologous recombination. A total of 14 hyg-resistant colonies were screened to confirm *71cR* disruption by PCR analysis using primer sequences shown in Table 2. Primers 71cR-Rev3 and 71cR-inv5’ that span the hyg-resistance cassette were used to screen the disruptants; with these primers the wt band is 400 bp and the band resulting from disruption is 2 kb (Fig 4A and 4B). A total of 12 transformants were confirmed as lacking the 400 bp wild type band. One had an ectopic integration of the split markers (#23) and one lacked evidence for integration of the split marker (#15) (Fig 4B). Eleven of the disruptants and the strain with ectopic integration of the split markers (#23) were then screened using a *71cR* locus-specific primer for the 5’ end outside the split marker sequence (71cR-Forw10) and a primer specific to hyg cassette sequence (Hyg-F2). Presence of the 1.7 kb fragment confirmed the correct location of split marker integration for 10 of the putative disruptants, and no band was seen on amplification of strain #23 (transformant with ectopic integration of the split markers) (Fig 4C). Finally, we screened transformants using a *71cR* locus-specific primer for the 3’ end outside the split marker sequence (71cR-DisR) and a second primer specific to the hyg cassette sequence (Hyg-R) (Fig 4D); all of the transformants were also confirmed to have a 715 bp band again confirming the correct location of the split marker integration. Through this analysis, 10 transformants (# 10, 13, 14, 16, 17, 18, 19, 20, 21, 24) were confirmed as being disrupted for *71cR*.

**Fig 5:**
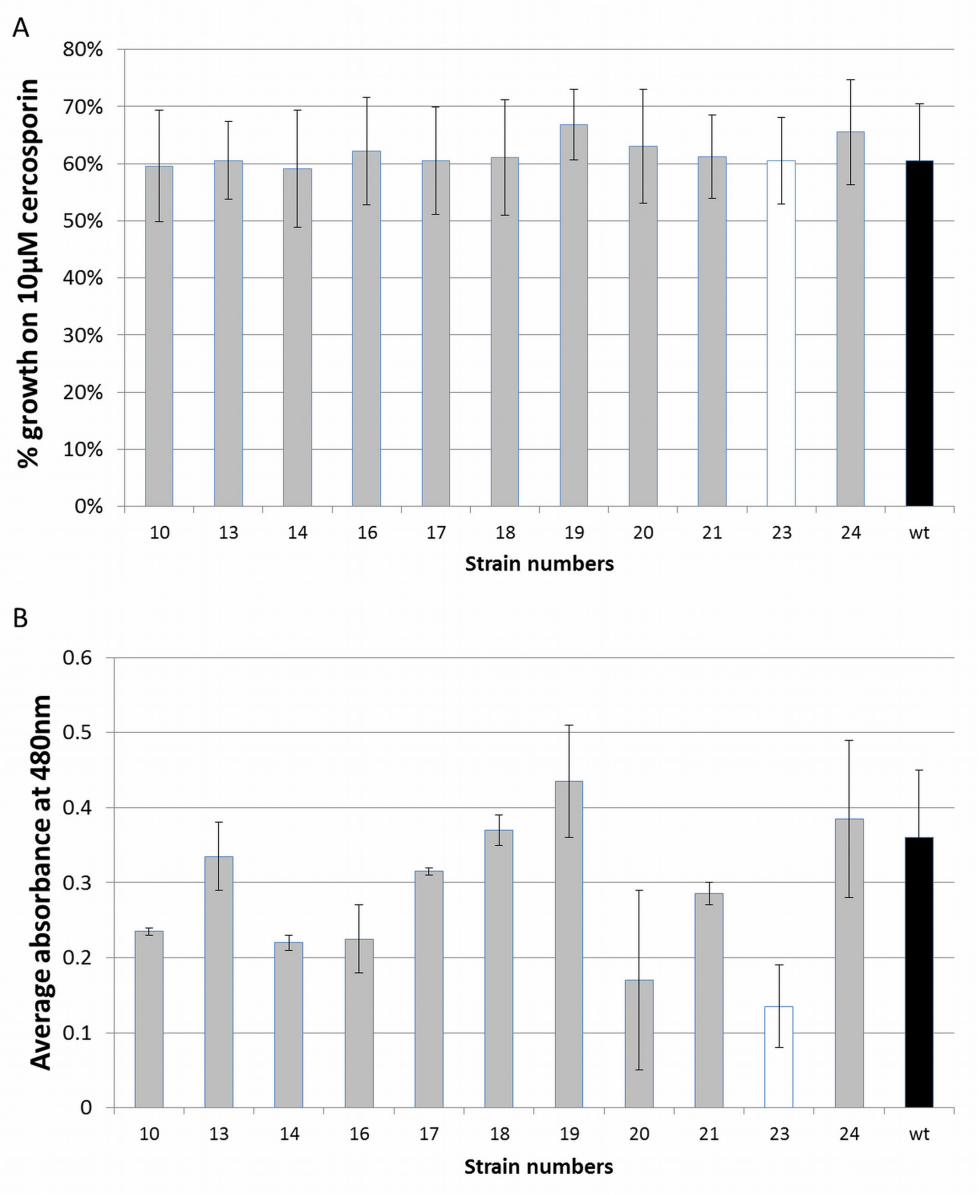
Phenotypic characterization of the *71cR* disruptants. **A.** Cercosporin resistance of *Cercospora nicotianae* wild type (black bar), *71cR-*disruptant strains (grey bars) and *71cR*-transformed, but non-disruptant (white bar). The cercosporin sensitive *crg1* mutant had 0% growth on cercosporin relative to control growth (not shown). Data are the result of two independent experiments with 4 replications each. Error bars represent standard error. **B.** Cercosporin production by *Cercospora nicotianae* wild type (black bar), *71cR-*disruptant strains (grey bars) and *71cR*-transformed, but non-disruptant strain (white bar). Cercosporin production was measured by extraction of mycelium with 5N KOH and measuring absorbance at 480 nm. Data are the results of two independent experiments with 2 replications each. Error bars represent standard error.

The ten 71cR-disruption strains were tested for cercosporin sensitivity by growing them on cercosporin-containing medium in the light. The wt, a transformed but non-disrupted strain (#23), and the cercosporin-sensitive *crg1* mutant [6] were used as controls. Results are shown in Fig 5A. Under the conditions of the assay, the *crg1* mutant does not grow on cercosporin (0% growth relative to the control [not shown]). There was no statistically significant difference in cercosporin resistance between the wt, the non-disrupted transformant (#23), and *71cR* disruptants in radial growth on cercosporin. As other resistance proteins such as ATR1 and CFP have shown a dual role in both cercosporin resistance and production [10, 12], *71cR* disruptants were also assayed for cercosporin production (Fig 5B) as compared to wt and the non-disrupted transformant #23. Cercosporin production varied between the different strains. However none of the *71cR* disruptants produced significantly less cercosporin than wt.

### Expression of transporters and cercosporin biosynthetic genes in *71cR* disrupted mutants under cercosporin toxicity

**Fig 6:**
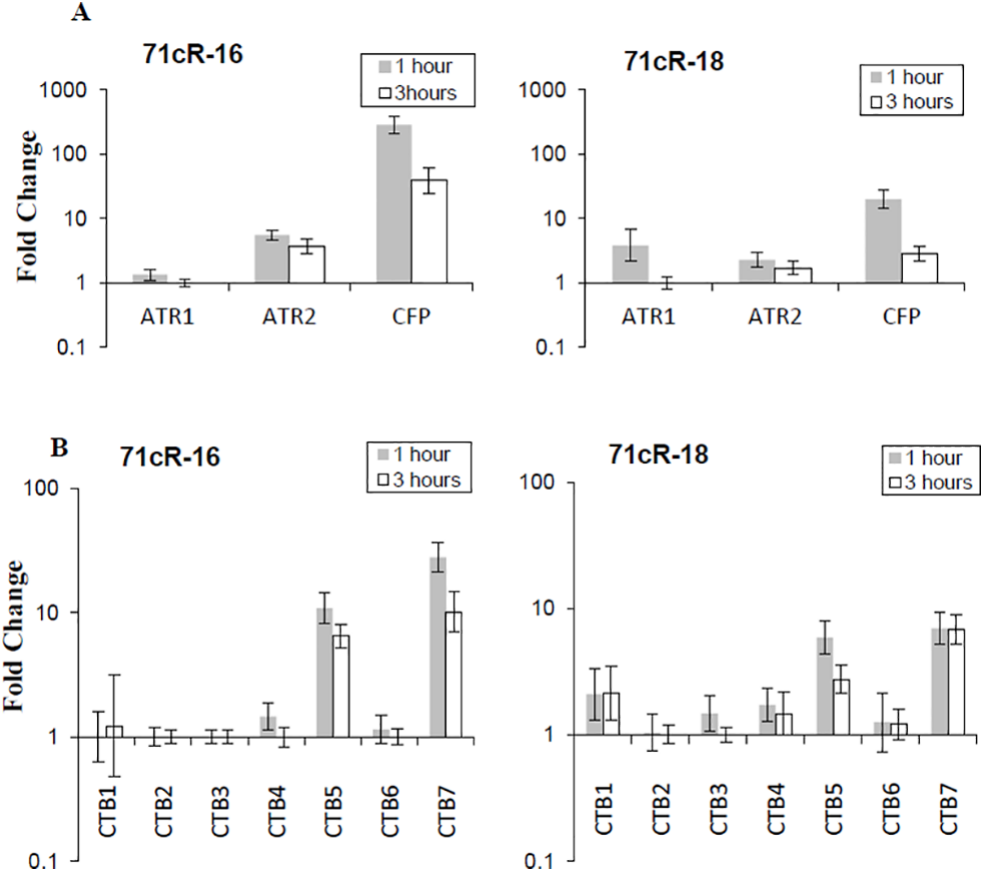
Quantitative RT-PCR analysis of gene expression of genes in the *C*. *nicotianae 71cR* mutant treated with cercosporin in the light. Two 71cR disrupted strains, 71cR-16 and 71cR-18, were tested. Each sample was normalized against the *C*. *nicotianae*-specific actin reference gene, and fold-change relative to the no-cercosporin control was calculated according to the 2^(-ΔΔC(T))^ method [21]. Data represent the mean of two independent experiments. Error bars represent 95% confidence intervals. **A.** Expression of 3 transporters in *71cR* disruptants #16 and #18. **B.** Expression of *CTB* genes in *71cR* disruptants #16 and #18.

Expression of *71cR* in *N*. *crassa* demonstrated that 71cR can provide cercosporin resistance, however, *71cR* disruption mutants of *C*. *nicotianae* were not more sensitive to cercosporin or produce less cercosporin than wt. Previous studies have suggested that *C*. *nicotianae* upregulates other resistance genes to compensate for resistance gene mutations [13]. We thus assayed for expression of additional library genes previously shown to impart cercosporin resistance (*CnATR1*, *CnATR2* and *CnCFP*) to determine if they are up-regulated in the *71cR* mutant background (Fig 6A). Two *71cR* disruptants (disruptant #16 and 18) were tested. Expression of *CnATR1* and *CnATR2* was not increased significantly in either of the disruptants. By contrast, *CnCFP* expression was strongly increased: 285 and 20 fold at 1 hour, and 40- and 3- fold at 3 hours in disruptants #16 and 18, respectively.

The subtractive library of putative cercosporin resistance genes also includes two genes from the cercosporin biosynthetic pathway: *CTB2* (*c*ercosporin *t*oxin *b*iosynthesis gene 2), encoding an *O*-methyltransferase, and *CTB5*, encoding an O_2_, FAD/FMN-dependent oxidoreductase [7,40]. Previous studies to test the role of each of the 8 biosynthetic pathway (CTB) gene products did not document a role in resistance [2], however we previously documented up-regulation of two of the CTB genes in response to cercosporin toxicity in *C*. *nicotianae* mutants deficient for a membrane transporter (ATR2) shown to play a role in cercosporin resistance [13]. Thus we assayed expression of all genes in the cercosporin biosynthetic cluster in the *71cR* disruption mutants under cercosporin toxicity. Two genes were significantly up-regulated: *CTB5* (found in the library) as well as *CTB7*, encoding a second FAD/FMN-dependent oxidoreductase (Fig 6B). Expression of *CTB2* and the other biosynthetic genes including the *CTB4* transporter gene were not altered. Up-regulation of *CTB5* and *CTB7* was less than that of *CFP* in both *71cR* disruptants induced with cercosporin toxicity. *CTB5* was induced 10- and 6-fold in disruptants #16 and 18, respectively, at 1 hour, and 6- and 3-fold, respectively, at 3 hours. For *CTB7*, fold-increase in disruptants #16 and 18 was, respectively, 28- and 10-fold at 1 hour and 6- and 7-fold at 3 hours. Interestingly, *CTB5* and *CTB7* are the same two *CTB* genes that are upregulated in response to cercosporin toxicity in the membrane transporter *atr2* mutants [13].

### Phylogenetic analysis of 71cR

*71cR* is an intronless 457 bp sequence. Blastp and tblastn searches on the NCBI database and tblastx with BLAST+ on the *Cercospora canescens* genome sequence revealed high homology to hypothetical proteins from many different fungi. The closest ortholog is in *Cercospora canescens* (ANSM00000000.1, 92% similar in 139 amino acids), with orthologs present in a wide range of fungi (S1 Table). Orthologs with high homology include ones from *Coniosporium apollinis Sterfl*. (XM_007783477.1, 84% similar in 151 amino acids), *Talaromyces stipitatus* (XM_002479737.1, 83% similar in 153 amino acids), and *Pyrenophora teres* (XM_003300623.1, 81% similar in 151 amino acids). None of the 130 closest relatives to the 71cR protein from different fungal strains found by the BLASTP search (the least similar one having 68% similarity in 133 amino acids) (S1 Table) have been characterized for function.

**Fig 7:**
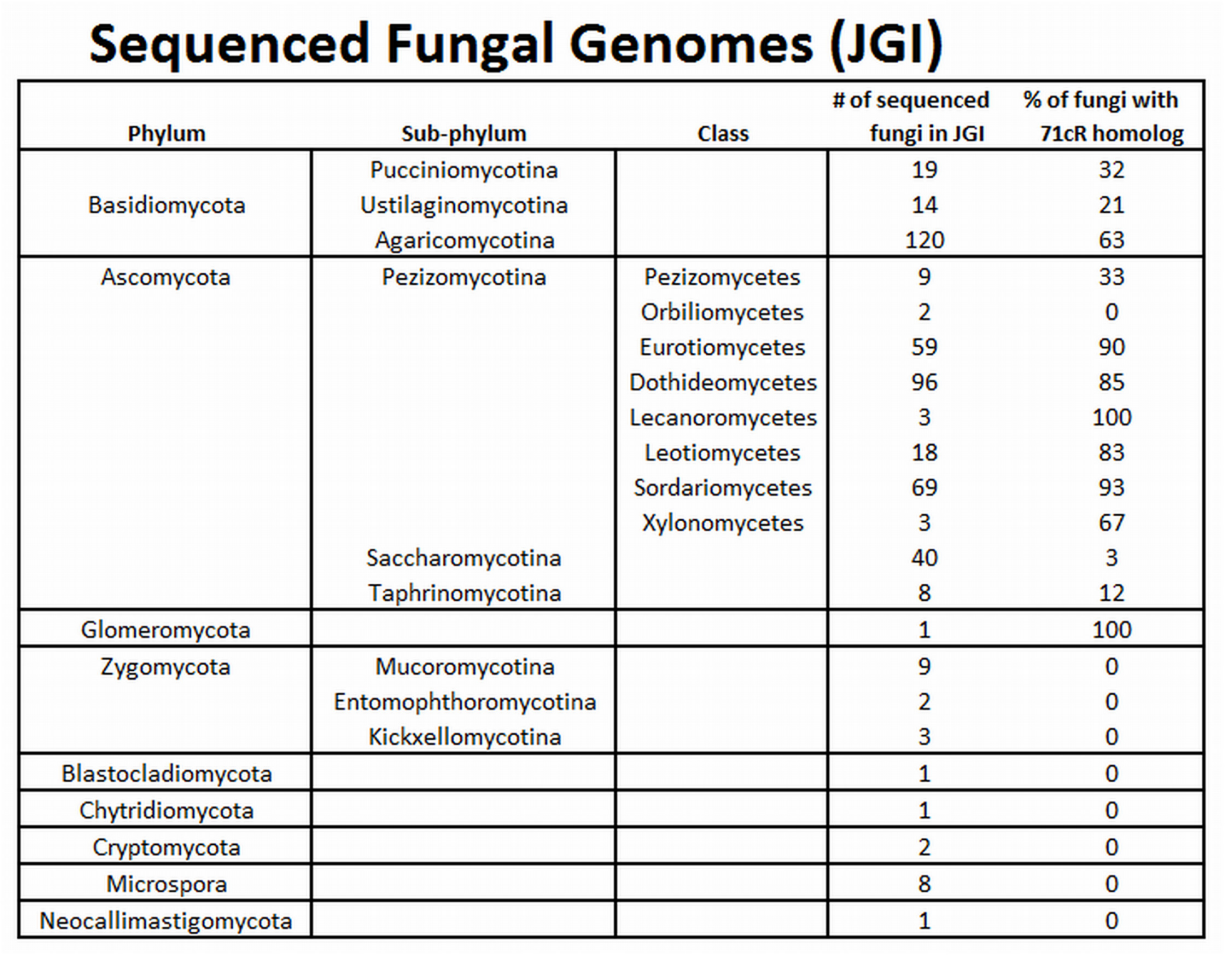
Distribution of 71cR orthologs among sequenced fungal species. For each genome sequence available in the JGI MycoCosm portal, tblastn was used to search the filtered model transcripts for 71cR orthologs. To the right of each taxon, the number of analyzed genome sequences is shown, along with the percent of genomes in the taxon for which a 71cR ortholog was identified.

To analyze the distribution of 71cR among fungal species, orthologs of the 71cR protein sequence were identified from sequenced fungal genomes in the JGI MycoCosm portal [33,34]. With one exception, 71cR orthologs were found in fungal genomes in all classes within the Dikarya (Basidiomycota and Ascomycota), including both mycelial and yeast species (Fig 7). Distribution was not ubiquitous, however, ranging from less than 5% to 100% of sequenced genomes within a taxonomic group. 71cR orthologs were especially common within the Ascomycota sub-phylum Pezizomycotina and the Basidiomycota sub-phylum Agaricomycotina. *Cercospora* spp. are in class Dothideomycetes within sub-phylum Pezizomycotina, and 85% of the 96 sequenced species within this class contained 71cR orthologs. While the *Cercospora canescens* genome contained a close ortholog, we were unable to identify an ortholog within the *Cercospora zeae-maydis* genome sequence. Distribution was less or lacking outside of the Dikarya. The one sequenced genome from the Glomeromycota (*Rhizophagus irregularis*) also had a 71cR ortholog, but 71cR orthologs were not found from other clades traditionally grouped into Zygomycota (such as Mucoromycotina and Kickxellomycotina) or from basal fungal lineages such as Neocallimastigomycota, Chytridiomycota, and Microsporidia (Fig 7). Due to the low number of sequenced genomes from these basal fungal lineages, however, it is not possible to definitively determine whether 71cR orthologs are completely absent within these taxa.

Because all orthologs of 71cR are hypothetical proteins, we searched for conserved domains in the protein sequence. A search on the Conserved Domain Database in the NCBI website [39] showed that a 131 amino acid portion of the 71cR protein aligns with a Nuclear Transport Factor 2 (NTF2-like) super-family domain. This result was confirmed using Pfam software [41] (version 2014_07), and the sequence was further identified as being in the DUF (*d*omain of *u*nknown *f*unction) 1348 family, one of the 24 families in the NTF2-like super-family. Of the 24 families in the NTF2-like super-family, 16 have known functions, and the remaining eight families, including DUF1348, are not characterized [41]. The DUF1348 family domain is present in 613 different UniProtKB proteins from 562 different organisms, 79 of which are in Eukarya and 534 of which are in Bacteria [41].

As the DUF1348 domain has not been characterized, relationships between the 71cR protein and proteins with known functions in the NTF2-like super-family were analyzed in an attempt to identify a putative function for 71cR. Maximum likelihood analysis was done for amino acid sequences of three DUF1348 domains including 71cR, as well as 54 proteins from different organisms representing each of the 16 characterized domains within the NTF2-like superfamily (S2 Fig). Some of the NTF2 families formed monophyletic clades. For example, the four scytalone dehydratase (SDH) sequences formed a monophyletic clade with a bootstrap value of 53%. However, sequences for most of the NTF2 families were polyphyletic, with little or no bootstrap support to cluster families within clades (S2 Fig). 71cR formed a clade with two other DUF1348 proteins from *Talaromyces* and *Pseudomonas* (S2 Fig) with a bootstrap value of 68%. The next closest domain was from a SnoaL-like polyketide cyclase from *Methylomicrobium album* from the NTF2 SnoaL1 domain family. Bootstrap support for the association with the SnoaL-like polyketide cyclase was very low (21%), however, thus no firm conclusions can be drawn about function from this analysis.

### Secondary and tertiary structure prediction of 71cR

**Fig 8:**
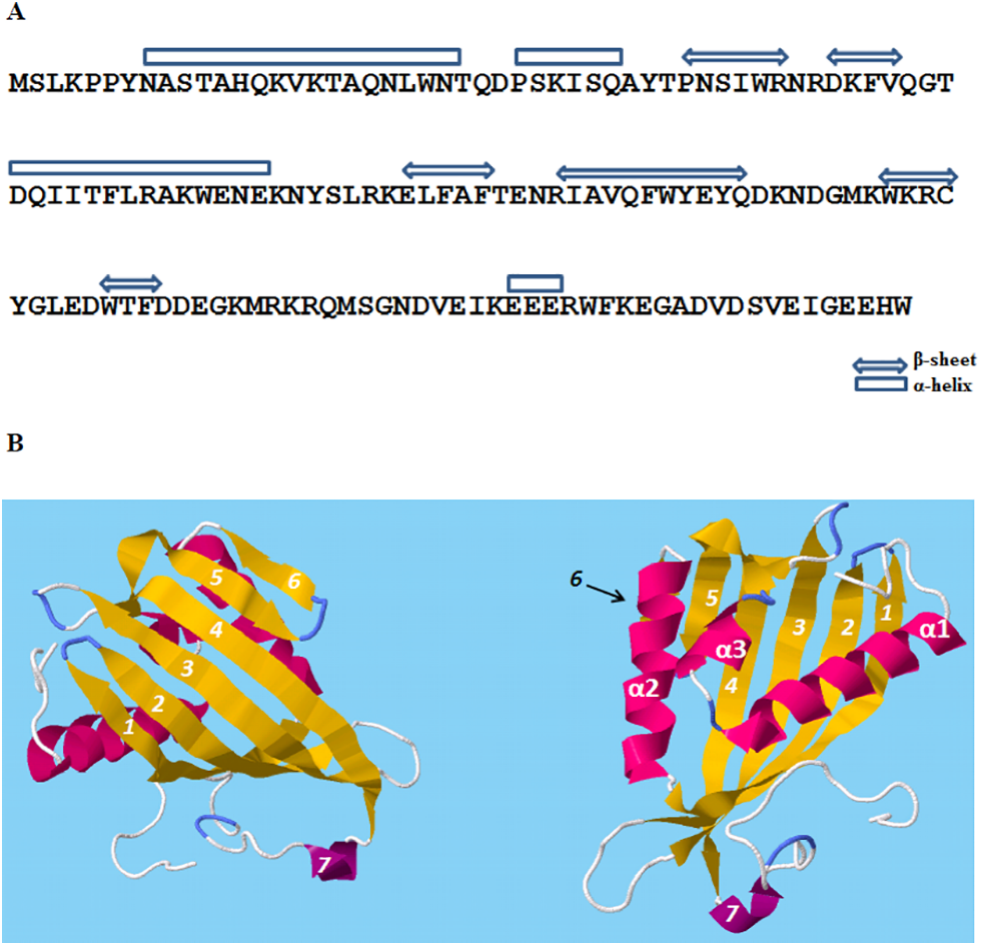
71cR secondary and tertiary structure predictions from Raptor software. **A.** Secondary structure showing 4 alpha helixes and 7 beta sheets. **B.** Predicted tertiary structure; the two figures represent the same structure from different angles. The beta sheets are numbered from 1–7, and alpha helixes are numbered from α1-α3. 71cR is predicted to have a half-barrel structure with 5 beta sheets enclosing the α1 helix. This protein is predicted to form a dimer to complete the barrel structure.

**Fig 9:**
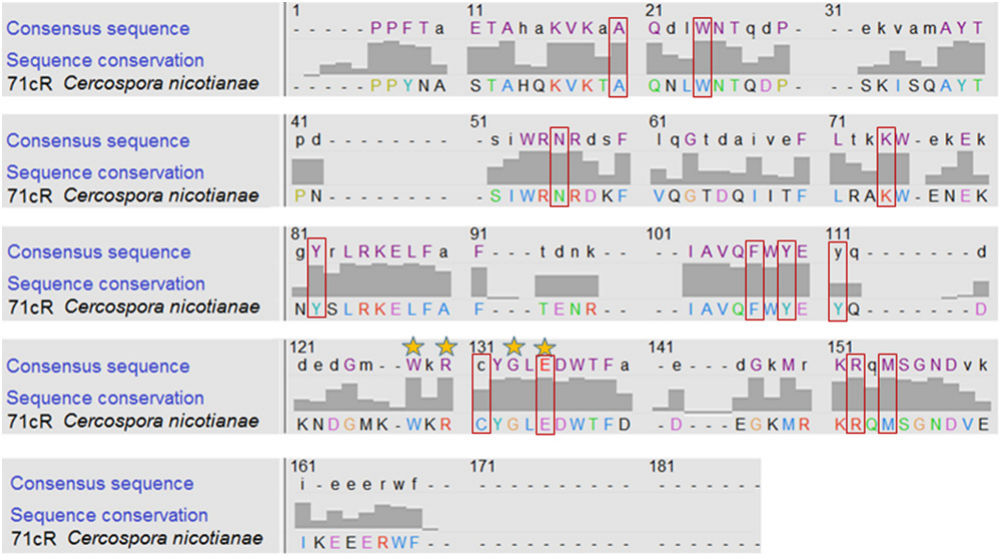
Alignment of 71cR amino acid sequence with the 130 amino acid sequences in S1 Table. Chimera 1.10.1 was used to display the degree of conservation for each residue within the alignment of the conserved domain [38]. 71cR *C*. *nicotianae* sequence is shown along with the consensus sequence from the whole dataset. The numbers above the amino acid residues indicate the positions within the sequence alignment. Color of each amino acid was determined according to the ClustalX [42] color scheme. The bars show the conservation between 71cR and all of the 130 sequences in S1 Table. Stars on top of the amino acids indicate the complete conservation of that sequence except the outgroup (E on position 135 is completely conserved throughout every single one including the outgroup). The red boxes around the letters show the ligand binding sites predicted by Raptor software.

As the phylogenetic analyses did not enable us to make conclusions about function, we analyzed secondary and tertiary structure of the 71cR amino acid sequence as these structures are important for their biological functions. The secondary and tertiary structure of the 71cR amino acid sequence was analyzed using Raptor software [35]. The secondary structure of 71cR is predicted to have 7 beta sheets and 4 alpha helices (Fig 8A). For the tertiary structure predictions, Raptor software predicted the best template to be Protein PFL_3262 from *Pseudomonas fluorescens* (2imj:A). The predicted model has a p-value of 2.96e^-11^. The predicted structure of 71cR protein is a half barrel structure with 7 beta sheets enclosing the alpha helix (Fig 8B). The two other alpha helices are positioned near beta sheets #4, 5, and 6 (Fig 8B). The predicted model also states that this protein forms a dimer. The Raptor server predicted that 71cR binds steroids, and identified 12 ligand binding sites for steroid binding (A19, W23, N43, K61, Y68, F86, Y88, Y90, C102, E106, R119, M121). The pocket multiplicity value predicted for steroid binding is 74, and we compared it to the pocket multiplicity of a known steroid delta-isomerase (Accession number: P07445); its pocket multiplicity was also 74, providing high support for 71cR binding to steroids. To determine whether the predicted ligand binding sites are conserved in 71cR, 130 of its closest orthologs (S1 Table) were aligned to identify conserved residues within the sequence (Fig 9). The 71cR sequence was mostly conserved throughout its length. All of the ligand binding sites found in the Raptor analysis except Y90 and C102 were shown to be conserved.

We then used the Dali server [36] which aligns tertiary structures of proteins. The crystal structure of the 71cR is not available. Thus we aligned the crystal structure of the closest ortholog with a crystal structure, protein PFL_3262 from *Pseudomonas fluorescens* (PDB ID: 2IMJ). This analysis predicts that the PFL_3262 tertiary structure aligns most closely with steroid delta-isomerase proteins, with z values of 14.1–15.4. This finding suggests that 71cR orthologs are most similar to steroid delta-isomerase proteins, a finding consistent with the Raptor software’s prediction of steroid binding. To further investigate this association, we re-ran the analysis with a known steroid delta-isomerase protein (Accession number: 3t8n). Z-scores between the steroid delta-isomerase protein (3t8n) and other steroid delta-isomerase proteins were higher, in the range of 20.4–25.7. We conclude that 71cR is similar to steroid delta-isomerase proteins, but is not this precise enzyme. In agreement with the similarity to steroid delta-isomerase proteins, which are localized in the cytosol, the Predict Protein server [37] identified no transmembrane helices or disulphide bridges in 71cR and predicted localization in the cytosol.

### Analysis of 71cR effect on cercosporin

Steroid delta-isomerase proteins catalyze isomerization reactions of steroids. We thus decided to test if 71cR may be catalyzing a reaction to degrade or modify the structure of cercosporin. We tested possible degradation by treating cultures of resistant *N*. *crassa 71cR* transformants (#379 and 380) and wt with cercosporin, harvesting the mycelium, and extracting and quantifying cercosporin from the mycelium and from the medium. We found no detectable differences in the amount of cercosporin recovered from the resistant transformants as compared to wt (data not shown), suggesting no degradation of cercosporin. In addition, we used confocal microscopy to generate λ-stacks to compare fluorescence emission spectra of the treated hyphae. We were unable to detect a difference between wild-type and resistant *N*. *crassa 71cR* transformants (data not shown). Thus we do not have evidence for degradation or modification of cercosporin by the *71cR* transformants.

## Discussion

The 185 EST sequences recovered from the subtractive library provided a large set of candidate gene products that may have a role in cercosporin resistance [7]. Among this list are three transporters, ATR1, ATR2 and CFP, that have been shown to have a role in resistance [10,12,13]. In this paper, we characterized genes in the library encoding hypothetical proteins. These were of particular interest to us, as resistance to ^1^O_2_ is poorly understood. To understand putative functions of the proteins encoded by the genes, the EST sequences were blasted against the NCBI protein database. Most of the ESTs aligned with orthologs with high similarity, however all of the orthologs identified encode proteins of unknown function. We then used the full length sequences of the closest orthologs to identify functional domains (Table 3). The results revealed diverse functional domains, most of which do not correspond to known mechanisms of cercosporin or ^1^O_2_ resistance.

To test the possible role of these hypothetical proteins in cercosporin resistance, we assayed for changes in gene expression when the *C*. *nicotianae* cercosporin-sensitive *atr1* transporter mutant was exposed to cercosporin toxicity. This mutant is sensitive due to a mutation in the ATR1 transporter involved in transport of cercosporin out of the cell [12], and has been used previously to identify genes that are upregulated under conditions of cercosporin toxicity and impart cercosporin resistance when expressed in sensitive fungi [13]. Only two of the 20 hypothetical protein-encoding genes, *24cF* and *71cR*, were significantly up-regulated. These results led us to further characterize them for a possible role in resistance.

To test the ability of these genes to increase cercosporin resistance, the cercosporin-sensitive fungus *N*. *crassa* was transformed with the complete gene sequences of *71cR* and *24cF* under the control of a constitutive promoter, and the resulting transformants were screened for increases in cercosporin resistance, an assay previously utilized to identify other cercosporin resistance genes [13,17]. Under the conditions of our cercosporin-sensitivity assay, growth of wt *N*. *crassa* is inhibited by about 70% whereas growth of the resistant *C*. *nicotianae* is inhibited by about 40%. No significant increase in resistance was seen in any of the *24cF* transformants tested, however significant increases in cercosporin resistance were found in some *71cR* transformants, with inhibition as low as 20%. Resistance of the *71cR* transformants varied, with some showing no increase. This variation might be explained by the random integration of the insert and different levels of transgene transcripts [43]. To confirm that the resistance seen in *71cR* transformants was due to the transgene, expression of the *71cR* transgene was tested from randomly picked transformants, two that showed significant resistance and two that did not. The results showed that transformants that showed increased resistance had high expression of the gene compared to the expression of the gene in the transformants that did not have increased resistance. Thus for 71cR transformants, expression of cercosporin resistance correlated with *71cR* gene expression. For 24cF, each of the transformants assayed had high levels of expression without imparting increases in resistance. Thus we concluded that 24cF alone cannot impart significant levels of cercosporin resistance. Given that *24cF* is upregulated under conditions of cercosporin toxicity, it is possible that it can play a role in resistance in combination with other genes. Our domain analysis (Table 3) showed that the protein has domains similar to those of phospholipid methyltransferases in the PEMT superfamily, but the precise function of this protein is unknown and further characterization will be needed to explore how it might contribute to resistance.

To further characterize the role of 71cR in cercosporin resistance, *71cR* was disrupted in wt *C*. *nicotianae*, and resistance to cercosporin was assayed. The resistance of the disruptants to cercosporin was not significantly different than the wt *C*. *nicotianae*. The cercosporin production in *71cR* disruptants was also compared to wt, and no difference in cercosporin production was observed. We hypothesized that the disruption of *71cR* may be compensated for by induction of other resistance genes. We thus assayed two of the *71cR* disruptants for expression of known resistance genes and for genes in the cercosporin biosynthetic pathway, when the disruptants were treated with cercosporin. Three genes, *CFP*, *CTB5* and *CTB7*, were found to be induced in the *71cR* disruptant mutants when they were exposed to cercosporin toxicity. CFP is an MFS transporter previously characterized to have a role in cercosporin resistance [10]. We have also shown that *CFP* is strongly induced by cercosporin toxicity in cercosporin-sensitive *atr1* and *atr2* mutants of *C*. *nicotianae* [13], thus CFP induction may be a general response of *C*. *nicotianae* against cercosporin toxicity, perhaps by facilitating export of the toxin out of the cells. The two *CTB* genes encode oxidoreductases found in the cercosporin biosynthetic cluster [2,44]. Oxidoreductase activity has been hypothesized to be involved in cercosporin resistance through reduction of cercosporin [2] and these genes are also induced in the cercosporin-sensitive *atr2* background [13]. Mutants for CTB5 and CTB7 have not been shown to be altered in resistance [40,44]; however, possible compensation by upregulation of other resistance genes in *ctb5* and *ctb7* mutants has not been investigated. Further studies with *ctb5* and *ctb7* disruption mutants need to be done to conclusively characterize a possible role in cercosporin resistance.

The ability of 71cR to impart cercosporin resistance in *N*. *crassa* led us to further characterize it. To identify the closest orthologs of 71cR, tblastn and blastp searches were done against the NCBI database, and 71cR was found to have high homology (up to 84% amino acid similarity) to hypothetical proteins from diverse fungi including members of both Ascomycota and Basidiomycota. To better understand species distribution within the fungal kingdom, we searched for orthologs of 71cR in each sequenced fungal genome from JGI using tblastn. This analysis showed that 71cR orthologs are widely distributed in the Basidiomycota and Ascomycota but that distribution is not ubiquitous within species in these groups. In the Ascomycota, orthologs were common in the Pezizomycotina, especially in the classes Dothidiomycetes (containing *Cercospora*), Eurotiomycetes, and Sordariomycetes. Orthologs of 71cR are also present in most fungal genomes in other subphyla in Ascomycota and Basidiomycota. *Rhizophagus irregularis*, the only sequenced genome from Glomeromycota, also had an ortholog of 71cR. However, 71cR orthologs were absent from sequenced genomes from other clades traditionally included in Zygomycota, as well as basal fungal lineages such as Chytridiomycota and Microsporidia (Fig 7). Although there are limited genomes available in JGI from these more basal lineages, the absence of this ortholog could indicate that 71cR evolved later in the evolutionary history of fungi.

As the most conserved orthologs to 71cR were not characterized for function, we used the Conserved Domain Database in the NCBI website [39] and Pfam [41] to identify domains. 71cR was shown to have an uncharacterized domain in the DUF1348 family in the NTF2 superfamily. The NTF2-like superfamily contains families with a common fold that results in a cone-like shape with a cavity inside. Although sharing a common fold, the proteins have very poor sequence similarity [45], thus explaining the lack of bootstrap support to cluster families within clades in our phylogenetic analysis (S2 Fig). The NTF2-like superfamily contains both enzymatically active and non-enzymatically active proteins. SnoaL polyketide cyclase, scytalone dehydratase, limonene-1,2-epoxide hydrolase and δ5-3-ketosteroid isomerase are examples of the enzymatically active group, which are typically intracellular [45]. Non-enzymatically active NTF2-like protein domains are mainly extracellular. The examples of proteins with non-enzymatically active NTF2-like domains are the C-terminus of calcium/calmodulin-dependent protein kinase II [46], Mba1, a putative ribosome-binding receptor [47], proteins involved in DNA transfer during bacterial conjugation [48], and immunity proteins in the bacterial polymorphic toxin systems [49]. Our phylogenetic analysis confirmed that 71cR formed a clade with other proteins containing a DUF1348 domain. The next closest characterized ortholog was a SnoaL-like polyketide cyclase from *Methylomicrobium album*, however, the bootstrap value for this relationship was extremely low (21%). Therefore, we were unable to draw any conclusions about the possible function of 71cR from the phylogenetic analysis.

Since we could not predict a function of 71cR based on sequence homology, we turned to characterization of the 71cR secondary and tertiary structures. Raptor software [35] predicted that the 71cR protein has antiparallel beta strands forming a half barrel structure with one of the main alpha helix structures buried inside this half barrel. The protein is predicted to form a dimer. Solvent Accessibility prediction [35] shows several ligand binding regions and predicted localization in the cytosol. With this information, the 71cR protein was predicted to have an enzymatically active group as it is intracellular. Furthermore, the Raptor server predicted that 71cR binds steroids (pocket multiplicity of 74), and predicted amino acid residues that would be important for ligand binding. We confirmed that most of the predicted ligand binding sites are conserved throughout the close orthologs of 71cR.

Further analysis of tertiary structure was done using the Dali server [36]. Using Dali, the 71cR protein aligned closely with steroid delta-isomerase proteins. Steroid delta-isomerase proteins are common in diverse organisms from bacteria to higher eukaryotes. They catalyze the isomerization of steroids by transferring protons in a two-step reaction [50], and are involved in both steroid biosynthesis [51] and steroid degradation pathways [52]. Although this was the closest association, z values were not high enough to conclude with confidence that 71cR is a steroid delta-isomerase, and it may catalyze a different reaction. We hypothesized that 71cR may impart resistance by degrading or altering the structure of the polyketide cercosporin molecule, but were unable to obtain evidence to support this hypothesis. It is interesting that so many fungi have orthologs to 71cR as most of these are not known to produce photoactivated perylenequinones or to be resistant to such compounds. It is possible that the enzymes, although all having DUF1348 domains, are very different. Alternatively, it is possible that they carry out similar reactions, but that the substrates are different in different species. Further research will be required to characterize the specific function of the 71cR protein as well as orthologs in other fungal species.

In summary, we have shown that the *71cR* gene, encoding a hypothetical protein, is upregulated in *C*. *nicotianae* in response to cercosporin toxicity, and that expression of this gene in the cercosporin-sensitive fungus *N*. *crassa* can impart cercosporin resistance. *C*. *nicotianae* disruption mutants are not more sensitive to cercosporin than wt, but other genes previously documented to play a role in resistance are up-regulated in these mutants upon exposure to cercosporin toxicity, suggesting that the fungus compensates for loss of resistance genes by up-regulation of other resistance mechanisms. Analysis of 71cR sequence, phylogeny, conserved domains, and secondary and tertiary structure identify the protein as having an NTF2-like superfamily domain with unknown function, to be intracellular, localized in the cytosol, to have enzymatic activity, and to have similarities to steroid delta-isomerase proteins. Current research is focused on expression of *71cR* in plants to determine if it has utility for engineering *Cercospora-* and cercosporin-resistant plants.

## Supporting Information

S1 Fig *Neurospora crassa* cercosporin resistance assay. Cercosporin resistance assay of *N*. *crassa* transformed with genes encoding hypothetical proteins from *Cercospora nicotianae*. Cultures grown under continuous light on Vogel’s medium supplemented with 10 μM cercosporin (right) and with 0.5% acetone (left) that was used to solubilize cercosporin. Top: *N*. *crassa* transformed with 71cR showing resistance to cercosporin. Middle: *N*. *crassa* transformant lacking resistance. Bottom: *N*. *crassa* wild type with colony margins at 21 hours (time used in resistance assay) marked on plate.
(TIF)

S2 Fig Phylogenetic tree of the conserved domains in 71cR in *C*. *nicotianae* and the proteins with known functions in the NTF2-like super-family. RAxML was used for maximum likelihood analysis of the sequences, which include the 71cR amino acid sequence, a hypothetical protein from *Talaromyces stipitatus* (XP_002479782.1) (a close ortholog of 71cR), and a *Pseudomonas fluorescens* protein (Protein Pfl_3262) in the same domain family (DUF1348), along with conserved domains of characterized proteins within the NTF2-like super family. Families within the NTF2-like superfamily are indicated in bold, followed by the name of the species from which the sequence was found. Domain abbreviations are: DUF1348, domain of unknown function 1348; RHBS, ring hydroxylating beta subunit; SnoaL, polyketide cyclase; LEH, limonene epoxide hydrolase; PHEN, phenazine biosynthesis protein; NTF2, nuclear transport factor 2; VirB8, type IV secretion assembly factor; WI12, wound induced protein; MTR2, nuclear pore RNA shuttling protein; Tim44, mitochondrial import protein; CDPK, Ca^2+^/calmodulin-dependent protein kinase; LBD, lumazine-binding domain; TRPEP, transpeptidase; MBA1, mitochondrial import protein; SDH, scytalone dehydratase. The red box indicates the clade containing the DUF1348 sequences. The 71cR protein from *C*. *nicotianae* is shown in red text. Bootstrap values for each relationship are presented on the tree, and the scale bar represents substitutions per site. The accession numbers with the species names where these proteins were found are included in S2 Table.
(TIF)

S1 Table Species names and Genbank accession numbers of closely related protein sequences to 71cR. This dataset was used in phylogenetics analysis of closely related sequences and analysis of conserved amino acid residues (Fig 9). Sequences are listed in order of similarity to the 71cR protein sequence.
(PDF)

S2 Table Domain function descriptions, species names, and Genbank accession numbers of proteins with known functions within the NTF2-like super-family. These protein sequences were used for phylogenetics analysis with conserved domains to identify the most closely related characterized ortholog of 71cR.
(PDF)
