# Supplementary figures and images for: Characterization of Cercospora nicotianae Hypothetical Proteins in Cercosporin Resistance
Source: PLoS One. 2015 Oct 16;10(10):e0140676. doi: 10.1371/journal.pone.0140676 (PMC4608573; doi:10.1371/journal.pone.0140676)

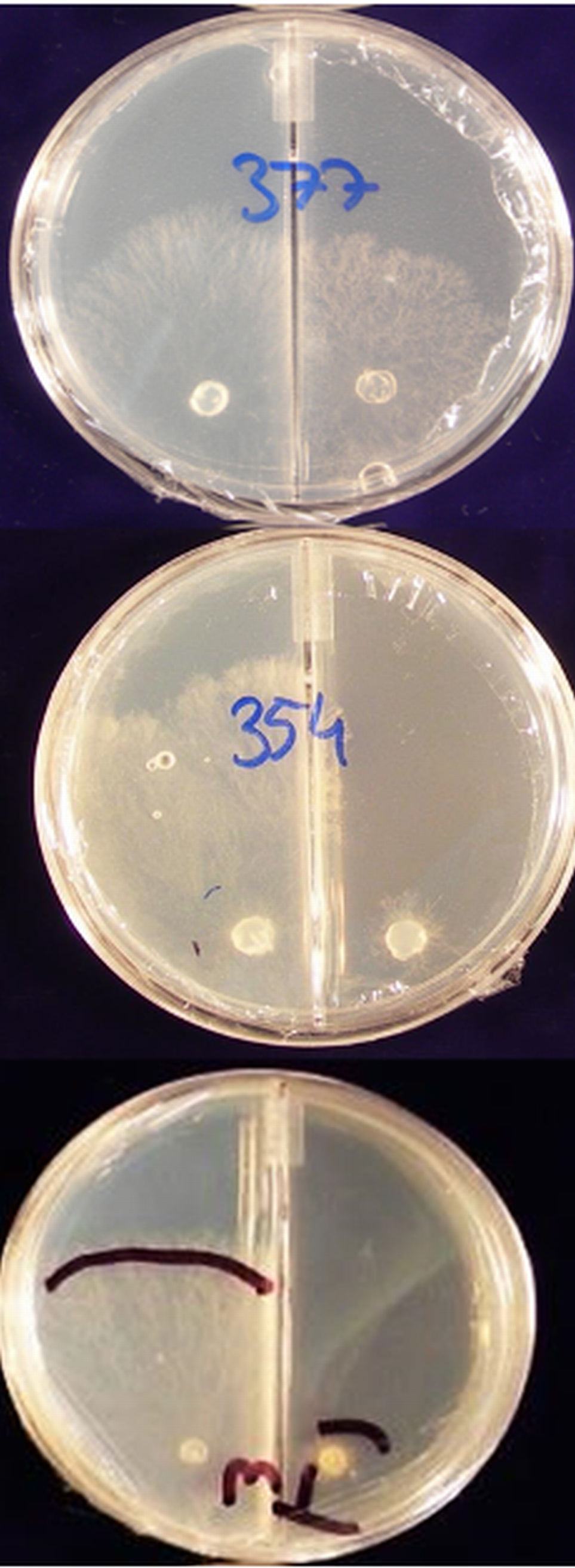

Supplement: S1 Fig — Cercosporin resistance assay of N. crassa transformed with genes encoding hypothetical proteins from Cercospora nicotianae. Cultures grown under continuous light on Vogel’s medium supplemented with 10 μM cercosporin (right) and with 0.5% acetone (left) that was used to solubilize cercosporin. Top: N. crassa transformed with 71cR showing resistance to cercosporin. Middle: N. crassa transformant lacking resistance. Bottom: N. crassa wild type with colony margins at 21 hours (time used in resistance assay) marked on plate. (TIF) [file pone.0140676.s001.tif]

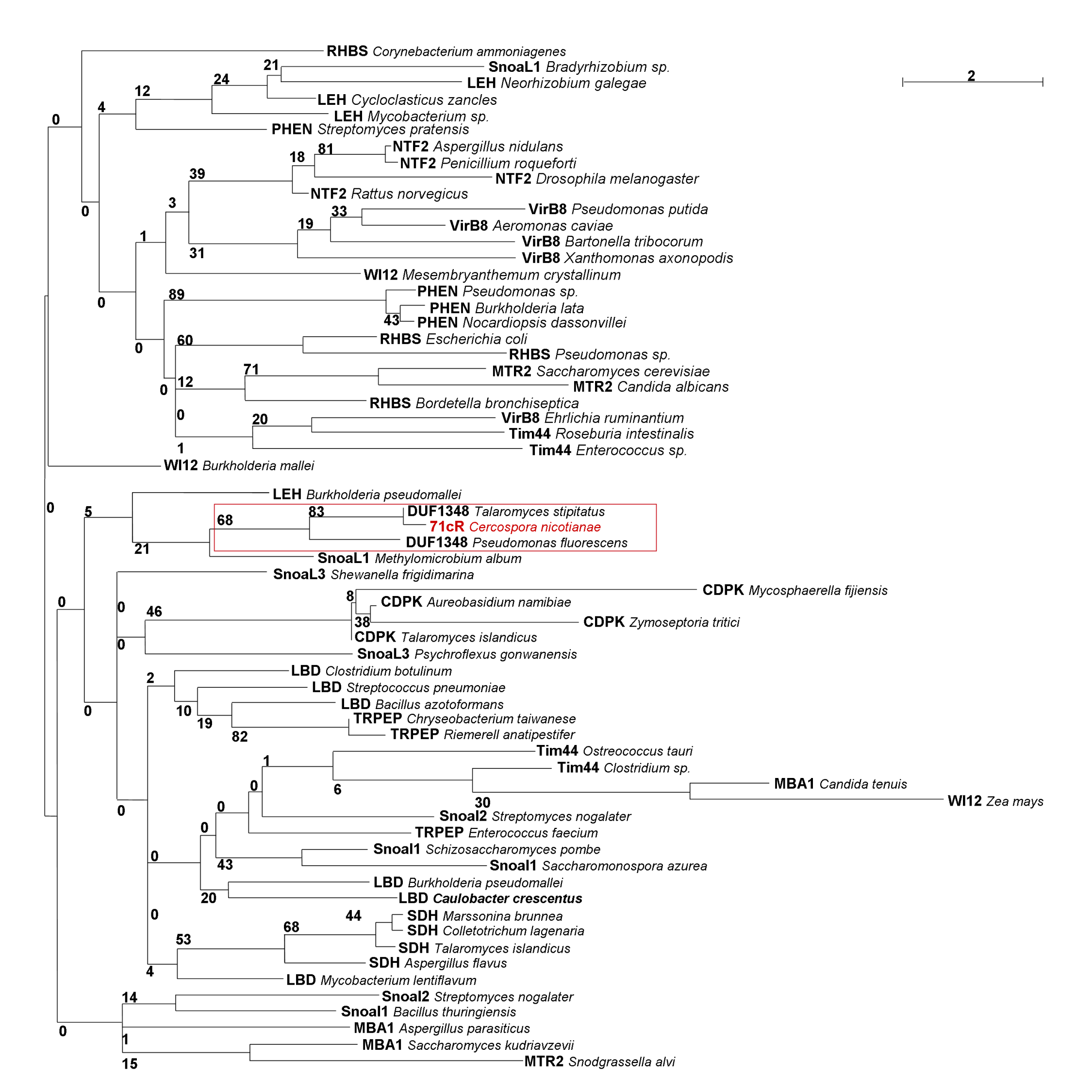

Supplement: S2 Fig — RAxML was used for maximum likelihood analysis of the sequences, which include the 71cR amino acid sequence, a hypothetical protein from Talaromyces stipitatus (XP_002479782.1) (a close ortholog of 71cR), and a Pseudomonas fluorescens protein (Protein Pfl_3262) in the same domain family (DUF1348), along with conserved domains of characterized proteins within the NTF2-like super family. Families within the NTF2-like superfamily are indicated in bold, followed by the name of the species from which the sequence was found. Domain abbreviations are: DUF1348, domain of unknown function 1348; RHBS, ring hydroxylating beta subunit; SnoaL, polyketide cyclase; LEH, limonene epoxide hydrolase; PHEN, phenazine biosynthesis protein; NTF2, nuclear transport factor 2; VirB8, type IV secretion assembly factor; WI12, wound induced protein; MTR2, nuclear pore RNA shuttling protein; Tim44, mitochondrial import protein; CDPK, Ca2+/calmodulin-dependent protein kinase; LBD, lumazine-binding domain; TRPEP, transpeptidase; MBA1, mitochondrial import protein; SDH, scytalone dehydratase. The red box indicates the clade containing the DUF1348 sequences. The 71cR protein from C. nicotianae is shown in red text. Bootstrap values for each relationship are presented on the tree, and the scale bar represents substitutions per site. The accession numbers with the species names where these proteins were found are included in S2 Table. (TIF) [file pone.0140676.s002.tif]
