# Supplementary material for: Characterization of Cercospora nicotianae Hypothetical Proteins in Cercosporin Resistance
Source: PLoS One. 2015 Oct 16;10(10):e0140676. doi: 10.1371/journal.pone.0140676 (PMC4608573; doi:10.1371/journal.pone.0140676)
Supplement: S1 Table — This dataset was used in phylogenetics analysis of closely related sequences and analysis of conserved amino acid residues (Fig 9). Sequences are listed in order of similarity to the 71cR protein sequence. (PDF) [file pone.0140676.s003.pdf]

S1 Table: Species names and Genbank accession numbers of closely related protein sequences to 71cR. This dataset was used in phylogenetics analysis of closely related sequences and analysis of conserved amino acid residues (Figure 9). Sequences are listed in order of similarity to the 71cR protein sequence.

Protein from:	Accession number
Cercospora nicotianae	AHN52028.1
Cercospora canescens	ANSM00000000.1
Coniosporium apollinis	XM_007783477.1
Talaromyces stipitatus	XM_002479737.1
Pyrenophora teres f. teres	XM_003300623.1
Cochliobolus sativus	XM_007696420.1
Bipolaris zeicola	XM_007708572.1
Bipolaris oryzae	XM_007684791.1
Baudoinia compniacensis	XM_007680086.1
Penicillium marneffei	XM_002143429.1
Pyrenophora tritici-repentis	XM_001935618.1
Aspergillus terreus	XM_001209907.1
Aspergillus clavatus	XM_001270301.1
Aspergillus niger	XM_001395869.1
Aspergillus oryzae RIB40	XM_001823820.1
Laccaria bicolor	XM_001877202.1
Penicillium chrysogenum Wisconsin 54-1255	XM_002566213.1
Stereum hirsutum	XM_007305684.1
Trametes versicolor	XM_008042907.1
Neosartorya fischeri	XM_001257415.1
Cladophialophora psammophila	XM_007751287.1
Endocarpon pusillum	XM_007789707.1
Capronia_coronata	XM_007729994.1
Schizophyllum commune	XM_003033985.1
Neofusicoccum parvum	XM_007580111.1
Sclerotinia sclerotiorum	XM_001593942.1
Myceliophthora thermophila	XM_003659899.1
Cladophialophora carrionii	XM_008729913.1
Chaetomium thermophilum var thermophilum DSM 1495	XM_006691885.1
Thielavia terrestris NRRL 8126	XM_003651594.1
Moniliophthora roreri	XM_007855888.1
Nectria haematococca mpVI 77-13-4	XM_003047637.1
Sordaria_macrospora k-hell	XM_003348336.1
Dichomitus squalens LYAD-421	XM_007363297.1
Cyphellophora europaea	XM_008720839.1
Eutypa lata	XM_007798760.1
Fibroporia radiculosa	XM_012326576.1
Magnaporthe oryzae	XM_003721003.1

Capronia epimyces	XM_007737565.1
Glarea lozoyensis	XM_008087450.1
Exophiala dermatitidis	XM_009155252.1
Setosphaeria turcica	XM_008023040.1
Metarhizium acridum	XM_007814472.1
Cladophialophora yegresii	XM_007756823.1
Neurospora tetrasperma	XM_009858003.1
Beauveria bassiana	XM_008598155.1
Neurospora crassa	XM_959108.3
Cordyceps militaris	XM_006668701.1
Colletotrichum gloeosporioides	XM_007283570.1
Togninia minima	XM_007915449.1
Moniliophthora perniciosa	XM_002389887.1
Cryptococcus neoformans var neoformans	XM_770297.1
Pestalotiopsis fici	XM_007830880.1
Colletotrichum fioriniae	XM_007590498.1
Verticillium dahliae	XM_009653173.1
Tremella mesenterica	XM_007005214.1
Trichoderma reesei	XM_006968205.1
Phanerochaete carnosa	XM_007397929.1
Exophiala dermatitidis	XM_009155251.1
Cryptococcus neoformans var grubii H99	XM_012194767.1
Gaeumannomyces graminis var tritici	XM_009229452.1
Ajellomyces capsulatus	XM_001538414.1
Aspergillus nidulans	XM_676870.1
Ajellomyces_dermatitidis	XM_002627550.1
Fomitiporia mediterranea	XM_007263484.1
Cryptococcus_neoformans_var_neoformans_JEC21	XM_571231.1
Punctularia_strigosozonata	XM_007389195.1
Phaeosphaeria_nodorum	XM_001797871.1
Selaginella moellendorfii	XM_002986561.1
Fusarium pseudograminearum	XM_009253654.1
Acanthamoeba_castellanii_str_Neff	XM_004336629.1
Aphanomyces_invadans	XM_008868897.1
Mycosphaerella fijiensis	XM_007922815.1
Aphanomyces astaci	XM_009829655.1
Saprolegnia parasitica	XM_012343185.1
Saprolegnia diclina	XM_008613522.1
Bipolaris victoriae	EUN23032.1
Macrophomina phaseolina	EKG13750.1
Rasamsonia emersonii	KKA18061.1
Byssochlamys spectabilis	GAD97874.1
Pyronema omphalodes	CCX10454.1
Oidiodendron maius Zn	KIM98742.1

Diplodia seriata	KKY14719.1
Fistulina hepatica	KIY50222.1
Bipolaris maydis C5	EMD96047.1
Aspergillus kawachii	GAA87712.1
Aureobasidium subglaciale	KEQ91447.1
Acremonium chrysogenum	KFH47914.1
Cylindrobasidium torrendii	KIY67985.1
Penicillium oxalicum	EPS31119.1
Rhizoctonia solani AG-1 IB	CCO31315.1
Penicillium solitum	KJJ25658.1
Fusarium oxysporum f. sp. cubense race 4	EMT66142.1
Rhizoctonia solani AG-1 IB	CEL54868.1
Fusarium oxysporum Fo5176	EGU87642.1
Verruconis gallopava	KIW09465.1
Laccaria amethystina LaAM-08-1	KIK08097.1
Fusarium oxysporum Fo47	EWZ46461.1
Rhizoctonia solani AG-8 WAC10335	KDN46588.1
Fusarium verticillioides 7600	EWG47493.1
Aureobasidium melanogenum CBS 110374	KEQ65606.1
Fusarium oxysporum f. sp. pisi HDV247	EXA43965.1
Aspergillus ruber CBS 135680	EYE90370.1
Fusarium oxysporum FOSC 3-a	EWZ00422.1
Penicillium expansum	KGO46950.1
Penicillium roqueforti FM164	CDM27309.1
Stachybotrys chartarum IBT 40288	KFA79299.1
Trametes cinnabarina	CDO75376.1
Penicillium italicum	KGO68117.1
Aspergillus fumigatus var. RP-2014	KEY83679.1
Aureobasidium namibiae CBS 147.97	KEQ72741.1
Rhizoctonia solani AG-3 Rhs1AP	EUC61276.1
Aspergillus rambellii	KKK12032.1
Verticillium alfalfae VaMs.102	XP_003007876.1
Talaromyces islandicus	CRG86219.1
Botrytis cinerea B05.10	XP_001551557.1
Botryobasidium botryosum FD-172 SS1	KDQ19526.1
Ceriporiopsis subvermispora B	EMD36508.1
Aureobasidium pullulans EXF-150	KEQ85392.1
Penicillium digitatum Pd1	EKV10058.1
Stachybotrys chlorohalonata IBT 40285	KFA64026.1
Exophiala oligosperma	KIW38269.1
Fusarium oxysporum f. sp. melonis 26406	EXK23376.1
Fomitopsis pinicola FP-58527 SS1	EPT04819.1
Sphaerobolus stellatus SS14	KIJ38456.1
Metarhizium majus ARSEF 297	KID96415.1

Fusarium graminearum	CEF74347.1
Metarhizium brunneum ARSEF 3297	KID79633.1
Exophiala mesophila	KIV91274.1
Exophiala aquamarina CBS 119918	KEF61283.1
Pseudogymnoascus pannorum VKM F-4515 (FW-2607)	KFY55947.1
Metarhizium anisopliae	KFG84629.1
