# Supplementary material for: Characterization of Cercospora nicotianae Hypothetical Proteins in Cercosporin Resistance
Source: PLoS One. 2015 Oct 16;10(10):e0140676. doi: 10.1371/journal.pone.0140676 (PMC4608573; doi:10.1371/journal.pone.0140676)
Supplement: S2 Table — These protein sequences were used for phylogenetics analysis with conserved domains to identify the most closely related characterized ortholog of 71cR. (PDF) [file pone.0140676.s004.pdf]

S2 Table: Domain function descriptions, species names, and Genbank accession numbers of proteins with known functions within the NTF2-like super-family. These protein sequences were used for phylogenetics analysis with conserved domains to identify the most closely related characterized homolog of 71cR.

Domains	Species	Accesion number
Ca2+/calmodulin-dependent protein kinase	*Talaromyces islandicus*	CRG91577.1
Ca2+/calmodulin-dependent protein kinase	*Aureobasidium namibiae CBS 147.97*	KEQ70053.1
Ca2+/calmodulin-dependent protein kinase	*Pseudocercospora fijiensis CIRAD86*	EME78224.1
Ca2+/calmodulin-dependent protein kinase	*Zymoseptoria tritici IPO323*	EGP92285.1
Limonene-1, 2-epoxide hydrolase	*Cycloclasticus zancles 7-ME*	AGS39004.1
Limonene-1, 2-epoxide hydrolase	*Neorhizobium galegae*	WP_046627776.1
Limonene-1, 2-epoxide hydrolase	*Burkholderia pseudomallei*	KKI72085.1
Limonene-1, 2-epoxide hydrolase	*Mycobacterium sp. UM_NZ2*	WP_046283582.1
Lumazine-binding domain	*Bacillus azotoformans MEV2011*	KEF37658.1
Lumazine-binding domain	*Streptococcus pneumoniae*	CKF20303.1
Lumazine-binding domain protein	*Clostridium botulinum 202F*	AIY78946.1
Lumazine-binding domain protein	*Mycobacterium lentiflavum*	CQD24850.1
Putative lumazine-binding	*Burkholderia pseudomallei*	CPH87736.1
MBA1-like protein	*Aspergillus parasiticus SU-1*	|KJK67754.1
MBA1-like protein	*Candida tenuis ATCC 10573*	EGV65914.1
MBA1-like protein	*Saccharomyces kudriavzevii IFO 1802*	EJT42540.1
NTF2-like N-terminal transpeptidase	*Chryseobacterium taiwanense*	KIC61686.1
NTF2-like N-terminal transpeptidase	*Riemerella anatipestifer RA-GD*	ADZ12144.1
NTF2-like N-terminal transpeptidase	*Enterococcus faecium TX1330*	EEI60277.1
Nuclear pore RNA shuttling protein Mtr2	*Saccharomyces cerevisiae*	CAA82029.1
Nuclear pore RNA shuttling protein Mtr2	*Snodgrassella alvi SCGC AB-598-O11*	KES10753.1
Nuclear pore RNA shuttling protein Mtr2	*Candida albicans*	P84148.1
Nuclear transport factor 2	*Rattus norvegicus*	NP_001007630.1
Nuclear transport factor 2	*Penicillium roqueforti FM164*	CDM28803.1
Nuclear transport factor 2	*Drosophila melanogaster*	AAS98195.1
Phenazine biosynthesis protein A/B	*Nocardiopsis dassonvillei*	ADH70409.1
Phenazine biosynthesis protein A/B	*Pseudomonas sp. GM17*	EJM14221.1
Phenazine biosynthesis protein A/B	*Streptomyces pratensis ATCC 33331*	ADW02923.1
Phenazine biosynthesis protein A/B	*Burkholderia lata*	ABB11682.1
Ring hydroxylating beta subunit	*Pseudomonas sp. HMSC05H02*	KJJ11284.1
Ring hydroxylating beta subunit	*Corynebacterium ammoniagenes DSM 20306*	EFG82184.1
ring hydroxylating beta subunit	*Bordetella bronchiseptica B20-10725633*	KDB66946.1
Scytalone dehydratase	*Colletotrichum lagenaria*	BAA13009.1
Scytalone dehydratase	*Talaromyces islandicus*	CRG86684.1
Scytalone dehydratase	*Aspergillus flavus AF70*	KJJ30121.1
Scytalone dehydratase	*Marssonina brunnea f. sp. 'multigermtubi'*	XP_007296032.1

SnoaL-like polyketide cyclase	*Saccharomonospora azurea NA-128*	EHY91179.1
SnoaL-like polyketide cyclase	*Bradyrhizobium sp. WSM471*	EHR01507.1
SnoaL-like polyketide cyclase	*Bacillus thuringiensis MC28*	AFU13283.1
SnoaL-like polyketide cyclase	*Methylomicrobium album BG8*	EIC29135.1
Snoal2-A Putative Hydroxylase	*Streptomyces Nogalater*	2GEX
Snoal3 -Ketosteroid Isomerase	*Shewanella Frigidimarina*	3BB9
Tim44-like domain	*Roseburia intestinalis XB6B4*	CBL12234.1
Tim44-like domain	*Clostridium sp. CAG:492*	WP_022091013.1
Tim44-like domain	*Ostreococcus tauri*	CEF99583.1
Tim44-like domain	*Enterococcus sp. 7L76*	CBL31786.1
VirB8 protein (plasmid)	*Aeromonas caviae*	YP_067832.1
VirB8 protein	*Ehrlichia ruminantium str. Welgevonden*	CAI26955.1
VirB8 protein	*Bartonella tribocorum CIP 105476*	CAK02009.1
VirB8 protein (plasmid)	*Pseudomonas putida*	YP_008994866.1
VirB8 protein (plasmid)	*Xanthomonas axonopodis pv. citri str. 306*	AAM39286.1
Wound-induced protein WI12	*Mesembryanthemum crystallinum*	AAD23999.1
Wound-induced protein WI12	*Burkholderia mallei*	KGC14241.1
Wound-induced protein WI12	*Zea mays*	ACG48358.1
